# Adaptive Genomic Backgrounds of Antimicrobial-Resistant *Salmonella enterica* from Chicken Sources: Virulence, Stress Response, and Genotype–Phenotype Discrepancy

**DOI:** 10.3390/antibiotics15100962

**Published:** 2026-09-29

**Authors:** Yihan Li, Vivienne Song, Andrew Wang, Shannon Chen, Yekai Xu, Wai Yung, Lucy Meng, Zuyi Huang

**Affiliations:** Department of Chemical and Biological Engineering, Villanova University, Villanova, PA 19085, USA

**Keywords:** antimicrobial resistance, *Salmonella enterica*, whole-genome sequencing, virulence genes, stress-response genes, genotype–phenotype discrepancy, principal component analysis, hierarchical clustering, logistic regression

## Abstract

Background: Antimicrobial-resistant *Salmonella enterica* from chicken sources is an important One Health concern because poultry can serve as a route for transmission of resistant foodborne pathogens. This study analyzed whole-genome sequencing data from 4611 chicken-associated *Salmonella enterica* isolates in the United States from the National Center for Biotechnology Information (NCBI) Pathogen Isolates Browser across seven antimicrobials: tetracycline, streptomycin, sulfisoxazole, ampicillin, ceftriaxone, nalidixic acid, and chloramphenicol. Methods: Principal component analysis, hierarchical clustering, and frequency-based bar-plot analysis were used to characterize gene-occurrence patterns and prioritize antimicrobial resistance (AMR), virulence, and stress-response genes. These prioritized genes were evaluated by logistic regression, and genotype–phenotype discrepancy analysis examined resistant isolates lacking expected antimicrobial-specific AMR genes using Fisher’s exact tests with Benjamini–Hochberg false discovery rate correction. Results: Resistant isolates shared broad genomic features: *mdsA* and *mdsB* were the most frequently detected AMR genes across all seven groups; virulence genes including *invA*, *iroB*, *iroC*, *sinH*, *pipA*, and *avrA* were repeatedly prominent; and *golS*, *golT*, and *asr* formed a common stress-response background. In the logistic regression models that predicted the probability that each *Salmonella enterica* isolate was resistant to a specific antimicrobial based on the presence or absence of selected genes, AMR-only models showed area under the receiver operating characteristic curve (AUC) values of 0.926–0.995, combined virulence and stress-response models showed AUC values of 0.826–0.990, and models combining all three gene categories showed AUC values of 0.935–0.998. Discrepant isolates were uncommon (0.06–2.10%), but enriched virulence and stress-response genes included recurrent *pco*, *sil*, *spv*, and *steB* signals across multiple antimicrobial groups. Conclusions: These findings identify resistance-associated genomic backgrounds involving virulence and stress-response genes and demonstrate their predictive value when incorporated into gene-based models. The enriched genes provide candidates for future genomic and experimental investigations to test whether they contribute functionally to antimicrobial resistance, persistence, or co-selection in poultry-associated Salmonella enterica.

## 1. Introduction

Antimicrobial resistance (AMR) is a major global public health and One Health concern, posing interconnected threats to human, animal, and environmental health. AMR can be a natural state for bacteria, and resistance mechanisms existed long before modern antimicrobial use. For example, in an isolated cave in New Mexico that had been separated for millions of years, scientists identified bacteria carrying many resistance mechanisms similar to those seen today, including drug-inactivating enzymes, target modification, and multidrug efflux pumps [1]. However, because of the misuse and overuse of antimicrobials, such as excessive use in human medicine and use in livestock production, AMR has become a major danger to healthcare and medicine [2]. It has been estimated that AMR could cause 10 million global deaths by 2050 if effective control strategies are not developed [3]. For foodborne pathogens, this problem is especially serious because resistant bacteria can move between animals, food products, the environment, and humans [4].

*Salmonella enterica* is a major foodborne pathogen and an important organism for AMR surveillance. *Salmonella* causes about 155,000 deaths worldwide every year [5]. In the United States, *Salmonella* is one of the leading causes of food poisoning and caused approximately 1.35 million infections every year (CDC, 2024) [6]. Among foodborne pathogens, *Salmonella* is commonly associated with chicken and poultry products [7]. Chicken is also one of the most frequently consumed meats in the United States (CDC, 2022 [8]). *Salmonella* can be transmitted to chickens through nonliving sources, such as feed, water, and litter, or through carrier animals such as rodents, insects, and wild animals [9]. After contamination, *Salmonella* can survive for long durations in both dry food products and wet environments [10]. Therefore, antimicrobial-resistant *Salmonella* from chicken sources is an important One Health concern [11].

The high AMR potential of *Salmonella* can be explained by several mechanisms, including *Salmonella* Genomic Island 1, the AcrAB-TolC tripartite efflux system, and the evolutionary and ecological pathway of *Salmonella* within zoonosis and the food chain [7,12]. Antimicrobial resistance can severely undermine the efficacy of medical treatment. Fewer antimicrobials are available to treat patients with severe *Salmonella* infections because of increasing AMR [7]. Therefore, it is necessary to better understand not only known AMR genes, but also other genomic features that may be linked with resistance, survival, and persistence [13,14].

Many AMR genes have been reported in *Salmonella*. Tetracycline resistance is often associated with *tet* genes, especially *tet(A)*, which can encode efflux pumps and reduce the effect of tetracycline on bacterial protein synthesis [15]. Sulfonamide resistance is commonly linked to *sul1* and *sul2*, which encode altered dihydropteroate synthase and allow folate synthesis to continue despite drug exposure [16]. Fluoroquinolone resistance is of concern because ciprofloxacin remains one of the commonly used antimicrobial agents for the treatment of various *Salmonella* infections [17]. Resistance can be related to *qnr* genes and mutations in DNA replication genes such as *gyrA*, which have been observed in retail meat isolates [18]. β-lactam resistance is often associated with β-lactamase genes such as *bla_CMY_* and *bla_CTX-M_*. The gene *bla_CTX-M-65_* is of particular concern because it provides resistance to ceftriaxone, one of the most important treatments for severe *Salmonella* infections [19]. In *Salmonella enterica* serotype Infantis, *bla_CTX-M-65_* has been found on multidrug-resistant plasmids that also carry other resistance genes such as *tet(A)* and *sul1*, and it has been observed in isolates from both chicken and human patients [19]. Surveillance programs such as the National Antimicrobial Resistance Monitoring System (NARMS) are therefore important for tracking resistant *Salmonella* in humans, retail meat, and food animals [20]. The National Center for Biotechnology Information has developed AMRFinderPlus and the Reference Gene Catalog to support accurate identification of AMR gene content [21]. Whole-genome sequencing (WGS) thus has made it easier to detect and analyze AMR genes [15,22,23,24,25].

Stress-response genes are one group of genes that may help explain broader bacterial adaptation. *Salmonella* must survive in many harsh environments, including poultry farms, food-processing facilities, dry food products, wet environments, and the host gastrointestinal tract [26]. In the food industry, chlorine and other disinfectants are commonly used to sanitize food and food-processing surfaces, but some *Salmonella* strains can survive the disinfection process [27]. During oxidative stress caused by chlorine disinfectants or reactive oxygen species, genes involved in stress response, Fe-S cluster formation, cysteine biosynthesis, biofilm formation, DNA protection, and iron homeostasis may be upregulated [27,28]. Genes such as *ycfR*, *dps*, *nrdM*, and *trxC* are associated with multiple stress-response, DNA-protection, and oxidoreductase processes [28]. Other important stress-related genes include *rpoS*, *oxyR*, chaperone genes, and the *clpXP* operon, which contribute to survival under osmotic, oxidative, and cold stress [27]. Metal- and biocide-associated genes may also be important. Genes such as *asr*, *arsD*, *merC*, *merP*, *merR*, *merT*, and *qacEdelta1* were commonly found in antimicrobial-resistant pathogens across several countries [29]. In addition, outlier outbreak genes in *Salmonella* identified by principal component analysis (PCA) and hierarchical clustering were mainly involved in stress response and included genes in the *ars* and *pco* operons, suggesting that stress-response genes may support survival in harsh conditions and outbreak success [30].

Virulence genes may also be connected with the adaptive background of resistant *Salmonella* via chromosomal and plasmid factors, including genes located in *Salmonella* pathogenicity islands. These genes contribute to adhesion, invasion, intracellular survival, biofilm formation, iron acquisition, and infection [31]. For example, *invA* is believed to trigger invasion into epithelial cells, and *spv* genes can increase the severity of enteritis and support infection at extraintestinal sites [32]. Several studies have found virulence genes from SPI-1, SPI-2, SPI-4, SPI-5, and plasmids in isolates from animals, foods, and humans [33,34,35]. In studies of NCBI Pathogen Detection data, virulence genes such as *fdeC*, *iha*, *iss*, *iutA*, *lpfA*, *sslE*, *ybtP*, and *ybtQ* were identified in several foodborne pathogens, including *Salmonella enterica* [36]. Although virulence genes are not the same as AMR genes, they may reflect a broader genetic background that supports colonization, persistence, or transmission [37].

Previous studies using the NCBI Pathogen Detection Isolates Browser have examined *Salmonella* genome diversity, AMR gene prevalence, plasmid content, and SNP-based clustering. For example, the pESI plasmid in emerging *Salmonella Infantis* has been reported to carry multiple AMR genes, virulence factors, and stress-response genes, including a mercury-resistance operon, which may improve persistence and spread [38]. AMR gene prevalence has also been shown to differ by country, source, and serotype, and NCBI SNP clustering has been used to support outbreak and epidemiologic investigations [39,40,41]. These studies show the value of WGS and NCBI Pathogen Browser data. However, many studies still focus mainly on known AMR determinants. Less work has systematically tested whether resistant *Salmonella* isolates from chicken sources also carry broader adaptive genomic features related to virulence, stress tolerance, metal resistance, biocide tolerance, environmental persistence, and food-chain survival.

This study addresses the gap between known AMR determinants and the broader genomic backgrounds associated with antimicrobial-resistant *Salmonella*. Although canonical AMR genes explain most resistant phenotypes, some isolates remain phenotypically resistant despite lacking the expected antimicrobial-specific determinants. Virulence and stress-response genes may not directly confer antimicrobial resistance, but they may contribute to colonization, survival, persistence, co-selection, or transmission under antimicrobial and environmental pressures. Therefore, it is important to determine whether these broader genomic features are associated with resistance and whether they are enriched in resistant isolates whose phenotypes are not explained by expected AMR genes.

To address this gap, chicken-associated *Salmonella enterica* isolates from the NCBI Pathogen Isolates Browser were analyzed using PCA [42,43,44], hierarchical clustering [45,46,47], and frequency-based analysis to prioritize AMR, virulence, and stress-response genes. Logistic regression [48,49] was then used to evaluate how these gene categories and their combinations distinguished resistance from susceptible isolates. Genotype–phenotype discrepancy analysis further identified resistant isolates lacking expected AMR determinants, and Fisher’s exact tests with Benjamini–Hochberg false discovery rate correction [50,51,52] were used to identify enriched virulence and stress-response genes. By integrating resistance prediction with genotype–phenotype discrepancy analysis, this study examines whether antimicrobial-resistant *Salmonella* is characterized only by canonical AMR determinants or also by a broader adaptive genomic background. Better characterization of these genomic patterns may support surveillance of resistant *Salmonella* in poultry production, help identify isolates of potential concern in the food chain, and ultimately contribute to food safety, consumer health, and One Health efforts to limit the spread of AMR.

## 2. Materials and Methods

### 2.1. Data Source and Dataset Structure

**Figure 1:**
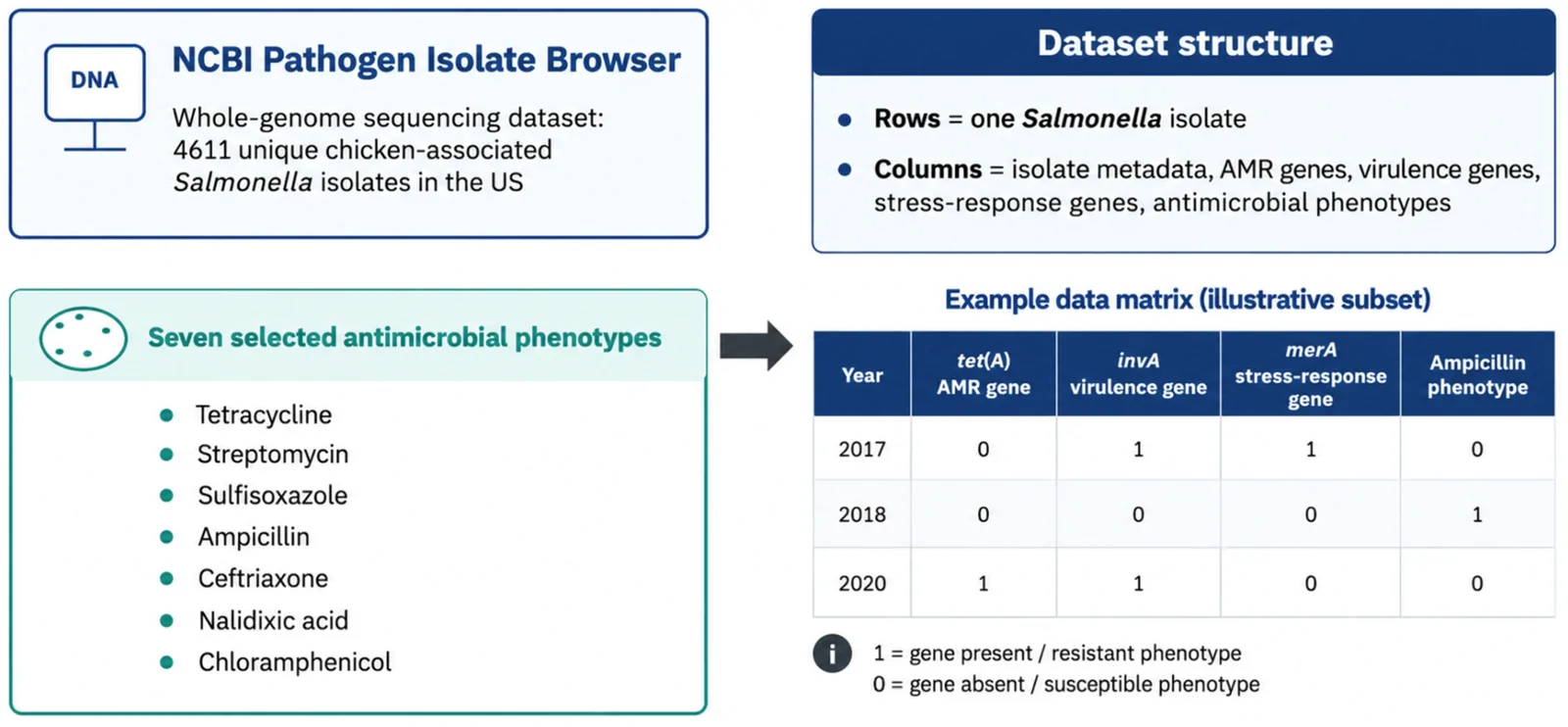
An overview of the materials extracted from the NCBI Pathogen Isolate Browser to study antimicrobial-resistant *Salmonella enterica* isolates from chicken sources in the USA.

This study used WGS information from the NCBI Pathogen Isolate Browser to study antimicrobial-resistant *Salmonella enterica* isolates from chicken sources in the USA. A total of 4611 chicken-associated *Salmonella* isolates sampled from 2015 to 2026 were included in the dataset. For each isolate, the data were organized as a matrix, with each row representing one *Salmonella* isolate and each column representing isolate metadata (Columns 1–4 for organism name, sampling location, collection date, and sample type), AMR genes (Columns 5–63), virulence genes (Columns 64–149), or stress-response genes (Columns 150–190), antimicrobial phenotypes (Columns 191–208). In this format, gene columns were coded based on whether each gene was detected in the isolate, which allowed us to compare gene occurrence patterns across resistant and susceptible groups. Seven antimicrobial phenotypes were selected for detailed analysis: tetracycline, streptomycin, sulfisoxazole, ampicillin, ceftriaxone, nalidixic acid, and chloramphenicol (Figure 1). These antimicrobials were chosen because they represent different antimicrobial classes and had enough resistant and susceptible isolates for comparison. For each antimicrobial, resistant isolates were first compared with susceptible isolates based on AMR genes, virulence genes, and stress-response genes.

### 2.2. Methods

**Figure 2:**
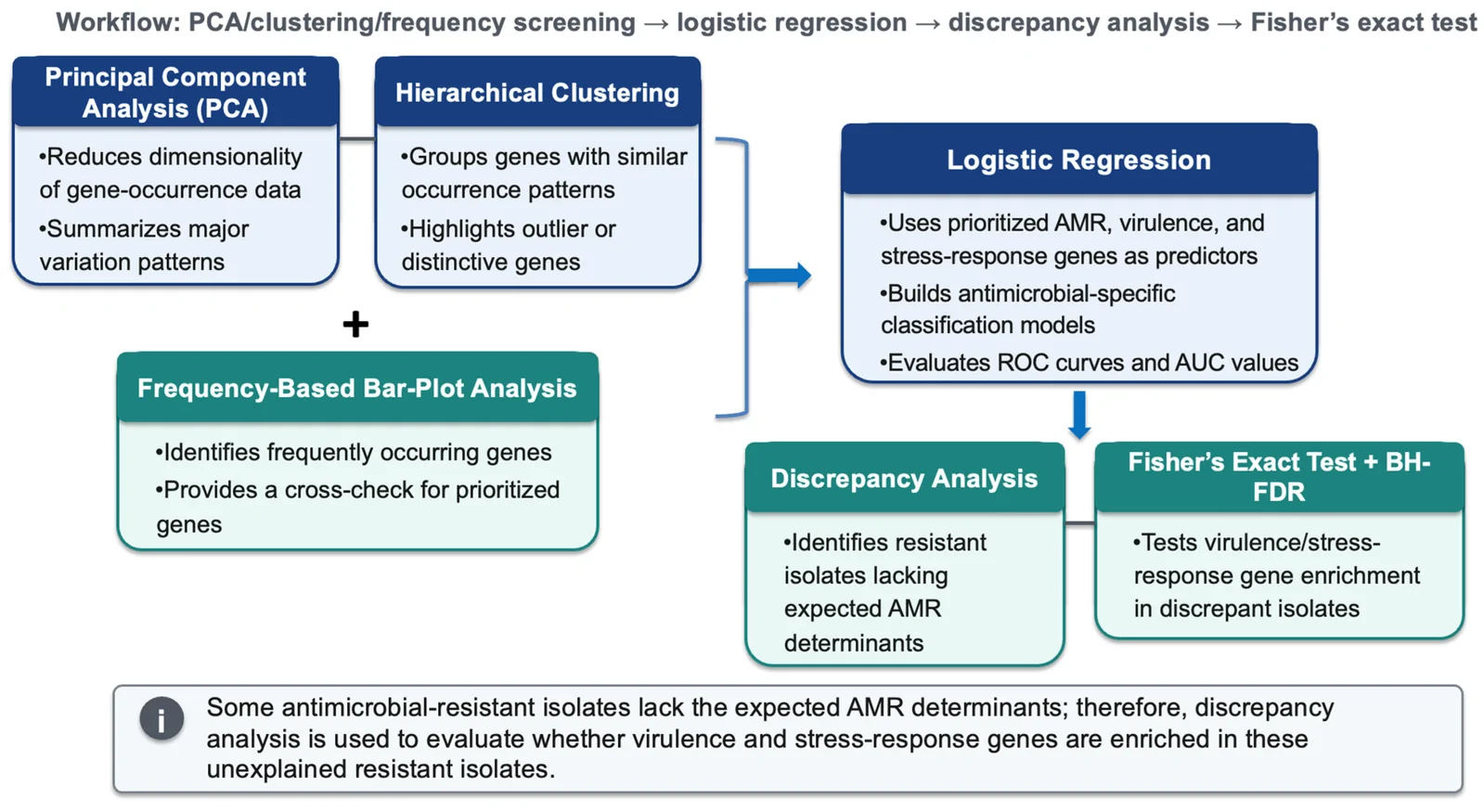
An overview of the analytical workflow for evaluating AMR, stress-response, and virulence genes in antimicrobial-resistant *Salmonella enterica* isolates from chickens in the United States.

Several complementary methods were used to identify important AMR genes, stress response genes, and virulence genes from *Salmonella enterica* isolates from chicken sources in the USA and to examine their occurrence patterns and statistical associations with phenotypic resistance to the seven selected antimicrobials. While the details of each of these methods will be detailed in the following subsection, an overview of them is given in Figure 2. PCA was first used to reduce the dimension of the gene-occurrence data and summarize major gene-pattern differences among isolates or antimicrobials [42]. Genes identified as outliers through PCA were further examined using hierarchical clustering to identify those located on distinct or outlying branches of the clustering tree [53,54]. The candidate genes identified through these two approaches were subsequently evaluated using frequency-based bar plots to determine their occurrence among resistant isolates. Genes located near the elbow point of the ranked frequency distribution, together with genes exhibiting relatively high occurrence frequencies, were prioritized as important genes for downstream analysis [53,54]. The important genes identified from clustering and frequency analysis were then used as inputs for logistic regression models [53]. These models evaluated how well different gene groups could predict antimicrobial resistance. Separate models were built using AMR genes only, virulence genes only, stress-response genes only, combining virulence and stress-response genes, and all gene groups together. Receiver operating characteristic (ROC) curves were used to evaluate how well each classification model distinguished antimicrobial-resistant *Salmonella enterica* isolates from susceptible isolates across different classification thresholds [55]. The area under the ROC curve (AUC) was used to compare model performance. This approach provided a systematic way to evaluate how AMR genes, virulence genes, and stress-response genes contribute to antimicrobial resistance patterns. Finally, discrepancy analysis [56] was performed because some phenotypically resistant isolates did not carry the expected antimicrobial-specific AMR genes. These isolates were defined as genotype–phenotype discrepant cases. Fisher’s exact test was then used to determine whether virulence or stress-response genes were enriched in these discrepant isolates. This analysis was designed to determine whether virulence and stress-response genes were disproportionately represented in resistant isolates lacking the expected antimicrobial-specific AMR determinants. Genes identified through this analysis were considered candidate markers of resistance-associated genomic backgrounds rather than established alternative resistance determinants. Additional genomic and experimental analyses would be required to determine whether these genes contribute functionally to the observed resistance phenotypes.

#### 2.2.1. Principal Component Analysis, Hierarchical Clustering, and Frequency Bar-Plot Analysis

This section uses antimicrobials as an example to illustrate the general procedure of principal component analysis, hierarchical clustering, and frequency bar-plot analysis used in this study. Before PCA was performed, the original isolate-level dataset was reorganized into an antimicrobial-by-gene matrix. In this matrix, individual antimicrobials were placed in the row position, and individual genes were placed in the column position. Each value represented the number of resistant isolates in which a given gene was detected for a given antimicrobial. This reorganized matrix allowed antimicrobial phenotypes to be compared based on their associated AMR, virulence, and stress-response gene profiles.

**Figure 3:**
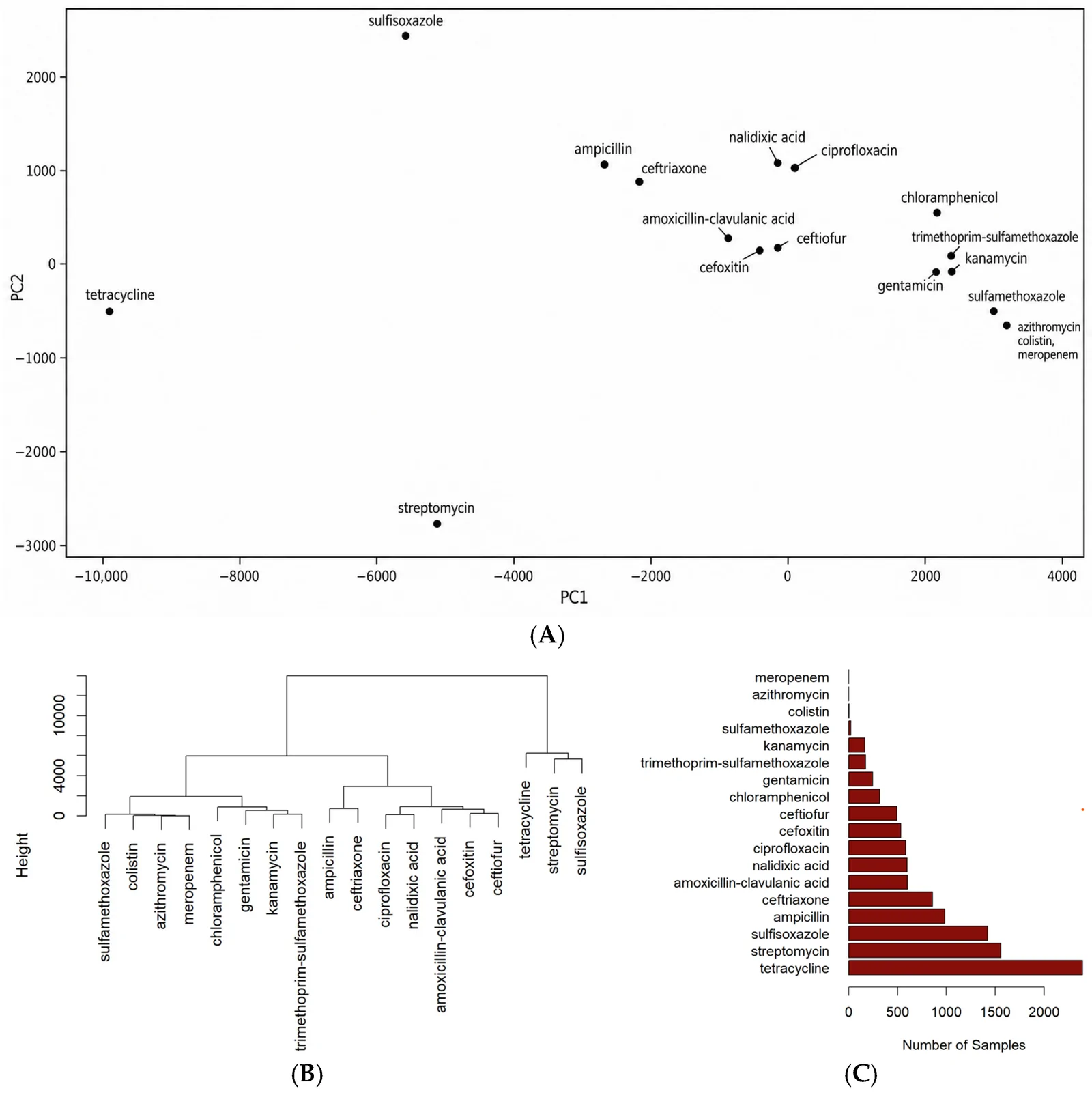
Illustrative overview of the principal component analysis, with PC1 and PC2 representing Principal Component 1 and 2 (**A**), hierarchical clustering (**B**), and frequency bar-plot analysis pipeline (**C**). Antimicrobials are used as an example to show how the original isolate-level dataset was reorganized into an antimicrobial-by-gene matrix, followed by PCA, hierarchical clustering, and frequency bar-plot analysis. This workflow was used to identify important antimicrobials and was later applied to AMR genes, stress-response genes, and virulence genes for downstream modeling and discrepancy analysis.

PCA was then applied to the reorganized matrix to reduce the dimensionality of the data. Because the original matrix contained many gene variables, direct visualization of antimicrobial relationships was difficult. PCA transformed these variables into a smaller number of principal components, where PC1 captured the largest source of variation and PC2 captured the second largest independent source of variation. In this study, PCA was used as an exploratory screening method to visualize how different antimicrobials were related based on their resistance-associated gene occurrence patterns. Antimicrobials located close together in the PCA plot were interpreted as having more similar gene profiles, whereas antimicrobials located farther away were considered to have more distinct profiles (Figure 3A).

After PCA, hierarchical clustering was applied to the PCA-reduced antimicrobial profiles to further evaluate the relationships among antimicrobials. While the PC1–PC2 projection provides a useful visual overview, overlapping points and limited two-dimensional resolution can make it difficult to determine quantitative relationships among antimicrobials. Hierarchical clustering therefore provided a complementary analysis by grouping antimicrobials with similar gene-occurrence patterns and displaying their relationships in a dendrogram (Figure 3B). The dendrogram provides an explicit representation of cluster membership and the relative separation among antimicrobial profiles, allowing relationships that may be difficult to distinguish in the two-dimensional PCA plot to be examined more clearly. Antimicrobials located on nearby branches were interpreted as having similar resistance-associated gene profiles, whereas antimicrobials located on long or separated branches were considered more distinct. Antimicrobials appearing on outlier branches were treated as potentially important candidates for further analysis.

Frequency histograms and bar plots were used in parallel with PCA and hierarchical clustering. PCA and clustering showed whether antimicrobials had similar or distinct gene-associated profiles, but they did not directly show how frequently resistance to each antimicrobial occurred. The frequency bar-plot therefore provided a practical cross-check by showing the number of resistant isolates for each antimicrobial (Figure 3C). An elbow-style approach [57] was used to guide antimicrobial selection by identifying a clear change point in the frequency distribution, where a smaller group of antimicrobials separated from the larger background group. Antimicrobial class representation was also considered so that the final selection covered multiple clinically and biologically relevant drug classes. Based on this combined approach, seven antimicrobials were selected for downstream analysis: tetracycline, streptomycin, sulfisoxazole, ampicillin, ceftriaxone, nalidixic acid, and chloramphenicol. These antimicrobials were then used for subsequent gene-level analyses, logistic regression modeling, and genotype–phenotype discrepancy analysis.

In addition to using antimicrobials as an illustrative example, the same PCA–clustering–frequency pipeline was applied at the gene level for each selected antimicrobial. For each antimicrobial, isolates resistant to that antimicrobial were first extracted as an antimicrobial-specific subset. Within this resistant subset, separate gene-occurrence matrices were then constructed for AMR genes, stress-response genes, and virulence genes. In these matrices, individual genes were placed in the row position, while isolates were placed in the column position. Each matrix entry represented whether a given gene was detected in a given resistant isolate. This structure allowed genes within each gene category to be compared according to their occurrence patterns across resistant isolates. Genes supported by PCA, hierarchical clustering, and frequency-based bar-plot analysis were selected as important genes for downstream analysis. Gene selection was performed sequentially by integrating PCA, hierarchical clustering, and frequency-based bar-plot analysis. First, PCA was used to identify genes exhibiting distinct or outlying occurrence patterns. These genes were further examined using hierarchical clustering to identify those located on distinct or outlying branches of the dendrogram. Genes identified as outliers by PCA and further supported by hierarchical clustering were retained as candidate genes for subsequent frequency-based analysis. Frequency bar plots were then used to rank these candidate genes according to their occurrence among resistant isolates. The elbow point was identified visually from the change in the slope of the ranked frequency distribution. Genes located near the elbow point, together with genes exhibiting relatively high occurrence frequencies, were prioritized as important genes for downstream analysis. Thus, the selection procedure integrated the three approaches sequentially rather than requiring genes to satisfy three independent numerical thresholds. No universal numerical frequency cutoff was applied across all antimicrobial-specific gene groups; instead, the observed elbow point and relative occurrence frequencies were used to guide the final selection within each group. These selected AMR, stress-response, and virulence genes were then used as inputs for logistic regression models later to evaluate how each gene group contributed to antimicrobial-resistance prediction. They were also used in genotype–phenotype discrepancy analysis to examine resistant isolates that lacked the corresponding expected AMR genes and to determine whether stress-response or virulence genes were enriched in these discrepant cases.

#### 2.2.2. Logistic Regression

Logistic regression was used to evaluate how the presence or absence of selected genes contributed to prediction of antimicrobial resistance [58]. This model is appropriate when the outcome variable has two categories. In this study, the outcome was the resistance phenotype of *Salmonella enterica* isolates for a specific antimicrobial, coded as 1 for resistant and 0 for susceptible or non-resistant. The data were organized in a row-by-column format, where each row represented one isolate and each column represented either the resistance phenotype or a binary gene variable. AMR genes, virulence genes, and stress-response genes were used as predictor variables, with 1 indicating that a gene was present and 0 indicating that it was absent. Accordingly, the logistic regression models were fitted separately for each antimicrobial. An isolate could therefore contribute to analyses for more than one antimicrobial when it exhibited multiple resistance phenotypes, but each model evaluated resistance to only one antimicrobial at a time. The resulting AUC values should therefore be interpreted as antimicrobial-specific predictive performance rather than as comparisons among independent isolated populations. In this study, predictive performance refers to the ability of a logistic-regression model containing a specified set of genes to distinguish resistant from susceptible isolates. The AUC values describe the performance of these multigene models and do not establish that every gene included in a model is independently predictive or directly confers resistance.

For each isolate, logistic regression generated a predicted probability between 0 and 1, where a higher value indicated a higher probability of resistance. When binary classification was needed, this probability could be compared with a threshold, such as 0.5, to classify isolates as predicted resistant or predicted susceptible. However, for ROC and AUC analysis, multiple thresholds were evaluated to measure the overall predictive performance of the model. In particular, the ROC curve plots sensitivity (true-positive rate) against the false-positive rate (1 − specificity) across classification thresholds, showing how well the model distinguishes resistant from susceptible isolates. The AUC summarizes this performance in a single value, with higher values indicating better discrimination and an AUC of 1 representing perfect discrimination. Logistic regression was selected because it constrains predicted probabilities within the 0–1 range, unlike linear regression, and because its coefficients provide an interpretable estimate of the direction and strength of association between gene occurrence and resistance. In this study, selected AMR genes, virulence genes, and stress-response genes were grouped in R as *amr_genes*, *virulence_genes*, and *stress_genes*, and these gene sets were tested separately and in combination to compare their contribution to antimicrobial-resistance prediction for each selected antimicrobial. The standard equation for logistic regression is shown below in Equation (1).

(1) $$\log(\mathrm{odds})=\log(\frac{p}{1-p})={\text{ß}}_{0}+{\text{ß}}_{1}x_{1}+{\text{ß}}_{2}x_{2}+\ldots +{\text{ß}}_{n}\;x_{n}$$

where ß_i_ represents the coefficient, *x* represents a specific gene, and *p* is the probability that an isolate is resistant.

While the logistic regression model predicts the probability that an isolate is resistant to a specific antimicrobial based on the presence of certain genes, odds ratio quantifies the change in odds of resistance with the presence of a gene. A larger odds ratio value could indicate that an isolate with a certain gene has higher odds of resistance to a specific antimicrobial while a smaller odds ratio value would indicate little association between the gene and resistance. The odds ratio formula is shown in Equation (2) below.

*odds ratio* = exp(ß_*i*_) (2)

where ß_i_ is the coefficient for a specific gene (i.e., Gene *i*). The *odds ratio* calculations provide a convenient method of interpreting the association between genes and resistance since gene coefficients by themselves are less intuitive to understand. 

#### 2.2.3. Discrepancy Analysis and Fisher Exact Test

Genotype–phenotype discrepancy analysis was performed to identify antimicrobial-resistant *Salmonella enterica* isolates in which the observed resistance phenotype could not be explained by the expected antimicrobial-specific AMR genes. For each of the seven selected antimicrobials, phenotypically resistant isolates were first extracted from the complete dataset. The expected AMR determinant sets were defined separately for each antimicrobial using the resistance-associated gene and mutation annotations represented in the dataset. Published genotype–phenotype studies of *Salmonella enterica* have established associations between antimicrobial resistance and major determinant groups [15,22,59,60,61], including *tet* genes for tetracycline, *aadA* and *aph* genes for streptomycin, *sul* genes for sulfonamides, β-lactamase genes for ampicillin and ceftriaxone, quinolone resistance-associated genes and *gyrA* mutations for nalidixic acid, and *floR*, *cat*, and *cmlA* genes for chloramphenicol. The complete antimicrobial-specific determinant sets used in the present analysis, including additional resistance-associated annotations represented in the original dataset, are provided in Table 1. They constitute the predefined operational criteria for genotype–phenotype discrepancy classification and do not imply that every listed annotation independently confers phenotypic resistance to the corresponding antimicrobial. For each antimicrobial, an isolate was classified as genotype–phenotype discrepant if it exhibited a resistant phenotype but none of the expected AMR determinants listed for that antimicrobial in Table 1 was detected in its genomic annotations. Conversely, a phenotypically resistant isolate was classified as carrying an expected AMR genotype if susceptibility tests indicated the resistance to the selected antimicrobial. The presence or absence of these determinants was determined using exact annotation-name matching, without automatically expanding gene-family names to include unlisted variants.

The genotype–phenotype discrepancy analysis was restricted to phenotypically resistant isolates because the dataset used for this analysis contained only isolates with a resistant phenotype. Consequently, the opposite type of discrepancy—an isolate carrying an expected AMR determinant but exhibiting a susceptible phenotype—could not be identified or evaluated in the present dataset. The analysis therefore focused on the primary research question of whether virulence and stress-response genes were enriched in resistant isolates lacking the expected antimicrobial-specific AMR determinants.

A resistant isolate was classified as an expected-AMR isolate when at least one of the expected antimicrobial-specific AMR determinants was detected. In contrast, a resistant isolate was classified as a genotype–phenotype discrepant isolate when none of the expected AMR determinants was detected despite the isolate having a resistant phenotype. Thus, the discrepancy analysis focused specifically on resistant isolates and divided them into two groups: resistant isolates containing one or more expected AMR genes and resistant isolates lacking all expected AMR genes.

The percentage of discrepant resistant isolates was calculated separately for each antimicrobial as follows in Equation (3).

(3) $$D_{\%}=(\frac{N_{\mathrm{disc}}}{N_{R}})\times 100\%$$

where *D*_%_ is the percentage of genotype–phenotype discrepant resistant isolates; *N_disc_* is the number of phenotypically resistant isolates lacking all expected antimicrobial-specific AMR genes; and *N_R_* is the total number of phenotypically resistant isolates for the antimicrobial being analyzed. This antimicrobial-specific approach was necessary because different antimicrobials have different expected resistance determinants. The resulting discrepancy percentage therefore represents the proportion of phenotypically resistant isolates whose resistance was not accounted for by the expected AMR genes included in the analysis.

After discrepant isolates were identified, Fisher’s exact test was used to determine whether individual virulence or stress-response genes occurred disproportionately in discrepant resistant isolates compared with resistant isolates carrying the expected AMR determinants. Fisher’s exact test was appropriate because the analysis involved categorical gene presence/absence data and because some genes occurred at low frequencies, producing small contingency-table cell counts for which a chi-square approximation may be inappropriate. For each virulence and stress-response gene, a 2 × 2 contingency table was constructed as shown in Table 2.

The null hypothesis was that the occurrence of a given virulence or stress-response gene was independent of whether a resistant isolate belonged to the discrepant or expected-AMR group. The alternative hypothesis was that gene occurrence differed between these two groups. For each gene, the percentage of isolates carrying the gene was calculated separately for the discrepant and expected-AMR groups. The difference in gene prevalence between the two groups was calculated via Equations (4) to (6).

*P*_D_ = a/(a + b) × 100% (4)

*P*_E_ = c/(c + d) × 100% (5)

Δ*P* = *P*_D_ − *P*_E_ (6)

where *P*_D_ is the gene prevalence in discrepant resistant isolates; *P*_E_ is the gene prevalence in resistant isolates carrying the expected AMR genes; and Δ*P* is the difference in gene prevalence between the two groups, expressed in percentage points. A positive Δ*P* indicates that the gene is more prevalent in the discrepant resistant group, whereas a negative Δ*P* indicates that the gene is less prevalent in that group.

An odds ratio was also calculated to describe the direction and magnitude of the association. An odds ratio greater than 1 together with a positive prevalence difference indicated that the gene occurred more frequently among discrepant isolates and was therefore enriched in the discrepant group. Because multiple virulence and stress-response genes were tested, *p* values were adjusted using the Benjamini–Hochberg false discovery rate (FDR) procedure. The FDR adjustment was applied within each gene group. Statistical significance was evaluated using the FDR-adjusted *p* value, while enrichment was defined by a positive prevalence difference and an odds ratio greater than 1. Importantly, enrichment of a virulence or stress-response gene in discrepant resistant isolates was interpreted as an association with the genomic background of the discrepant group rather than evidence that the gene directly confers resistance. Such genes may mark alternative resistance-associated backgrounds, linked mobile genetic elements, stress-adaptation systems, or other genomic features for further investigation.

## 3. Results

### 3.1. AMR, Virulence, and Stress-Response Gene Profiles in Salmonella enterica Isolates Resistant to the Seven Selected Antimicrobials

The gene-frequency rankings presented in this section describe the occurrence of annotated AMR, virulence, and stress-response genes among isolates resistant to each antimicrobial. High occurrence within a resistant group does not, by itself, demonstrate that an individual gene predicts resistance or directly confers resistance to the antimicrobial defining that group.

#### 3.1.1. AMR Gene Profiles in *Salmonella enterica* Isolates Resistant to the Seven Selected Antimicrobials

Table 3 provides a comparative summary of the AMR genes identified by PCA, hierarchical clustering and the frequency bar-plot analysis in *Salmonella enterica* isolates resistant to tetracycline, streptomycin, sulfisoxazole, ampicillin, ceftriaxone, nalidixic acid, and chloramphenicol. For each antimicrobial, genes are arranged from the highest to the lowest occurrence among resistant isolates. The ranking is therefore antimicrobial-specific: Rank 1 represents the most frequently detected AMR gene within that resistant-isolate group, Rank 2 represents the second most frequent gene, and so forth. This organization allows the major AMR-gene patterns to be compared across all seven antimicrobials in a single table while avoiding the need to present a separate frequency bar plot for every antimicrobial.

Across all seven antimicrobial groups, *mdsA* and *mdsB* ranked first and second, respectively. *tet(A)* was also frequently detected, ranking third for tetracycline, sulfisoxazole, ampicillin, and ceftriaxone; fifth for nalidixic acid and chloramphenicol; and sixth for streptomycin. Among tetracycline-resistant isolates, *tet(A)* ranked third, followed by *aph(3″)-Ib*, *aph(6)-Id*, *tet(B)*, *sul2*, and *sul1*. *aadA1*, *gyrA_D87Y*, and *bla_CMY-2_* were also among the higher-ranked genes. Among streptomycin-resistant isolates, *aph(3″)-Ib* and *aph(6)-Id* ranked third and fourth, followed by *tet(B)* and *tet(A)*, while *aadA1* ranked seventh. For sulfisoxazole-resistant isolates, *tet(A)* ranked third, followed by *sul2* and *sul1* at ranks four and five. Other frequently detected genes included *aadA1*, *gyrA_D87Y*, *bla_CMY-2_*, *aph(3′)-Ia*, and *aac(3)-IVa*. Ampicillin- and ceftriaxone-resistant isolates showed similar rankings: *tet(A)* ranked third, *bla_CMY-2_* fourth, and *sul2* fifth in both groups. In ampicillin-resistant isolates, *sul1*, *gyrA_D87Y*, and *bla_CTX-M-65_* ranked sixth through eighth, whereas in ceftriaxone-resistant isolates, *gyrA_D87Y*, *bla_CTX-M-65_*, and *sul1* occupied ranks six through eight. For nalidixic acid-resistant isolates, *gyrA_D87Y* ranked third, followed by *sul1* and *tet(A)* at ranks four and five. Other highly ranked genes included *aadA1*, *aac(3)-IVa*, *aph(4)-Ia*, *floR*, and *bla_CTX-M-65_*. For chloramphenicol-resistant isolates, *floR* ranked third, followed by *gyrA_D87Y* and *tet(A)* at ranks four and five. *aph(4)-Ia*, *aac(3)-IVa*, *sul1*, *aadA1*, and *bla_CTX-M-65_* were also among the ten most frequent genes, while *cmlA5* appeared lower in the ranking.

#### 3.1.2. Virulence Gene Profiles in *Salmonella enterica* Isolates Resistant to the Seven Selected Antimicrobials

Table 4 summarizes the virulence-gene frequency profiles for isolates resistant to the seven selected antimicrobials. A common set of frequently detected genes included *invA*, *iroB*, *iroC*, *sinH*, *pipA*, *avrA*, *hscC*, *envF*, *stfA*, *allD*, *icmF*, *gip*, *lpfB*, *sciR*, *lsrF*, *fljA*, *stiA*, and *stjB*, although their rank order differed among antimicrobial groups. In tetracycline-resistant isolates, *invA*, *iroB*, *iroC*, *sinH*, *pipA*, and *avrA* occupied the six highest ranks, followed by *hscC*, *envF*, *stfA*, *allD*, *icmF*, *gip*, *lpfB*, *sciR*, and *lsrF*. In streptomycin-resistant isolates, *invA* ranked first, followed by *iroC*, *sinH*, *iroB*, and *pipA*, with *icmF* and *avrA* also among the higher-ranked genes. The lower-ranked streptomycin profile additionally included *stkA*, *steB*, *iucA*, and *iucB*. For sulfisoxazole-resistant isolates, *iroB* and *sinH* ranked first and second, followed by *invA* and *iroC*; *lsrF*, *icmF*, *pipA*, and *avrA* were also among the highest-ranked genes. Ampicillin- and ceftriaxone-resistant isolates showed similar upper-ranking profiles, with *iroB* and *sinH* ranking highest, followed by *avrA*, *invA*, *iroC*, *pipA*, and/or *lsrF*. Nalidixic acid-resistant isolates showed a similar ordering, with *iroB*, *sinH*, *avrA*, *invA*, *iroC*, *lsrF*, and *pipA* among the highest-ranked genes. Chloramphenicol-resistant isolates showed a different rank order. *avrA* ranked first, followed by *fljA*, *hscC*, and *icmF*. *invA*, *iroB*, and *iroC* also remained among the more frequently detected genes, followed by *lsrF*, *pipA*, and *sinH*. Additional genes detected in this group included *stfA*, *lpfB*, *sciR*, *envF*, *ripR*, *sspH2*, *stm0859*, *allD*, *gip*, *stm0854*, *pagO*, *sseK2*, *pagK*, *stiA*, *safB*, *tcfA*, *tinR*, *ybtQ*, *ybtP*, and *stjB*.

#### 3.1.3. Stress Response Gene Profiles in *Salmonella enterica* Isolates Resistant to the Seven Selected Antimicrobials

Table 5 summarizes the stress-response gene frequency profiles for isolates resistant to the seven selected antimicrobials. *golS*, *golT*, and *asr* ranked first through third in all seven groups. In most groups, these genes were followed by *merR*, *merP*, and *merA*, while the positions of *arsD*, *arsR*, and *qacEdelta1* varied among antimicrobials. In tetracycline-resistant isolates, *golS*, *golT*, and *asr* were followed by *merR*, *merP*, *merA*, *arsD*, and *qacEdelta1*. Additional *mer* genes, including *merT*, *merC*, *merD*, and *merE*, occurred in intermediate positions, with *sil* and *pco* genes appearing lower in the ranking. In streptomycin-resistant isolates, *arsD* ranked fourth, followed by *silE*, *silB*, *silC*, *silF*, *silS*, *silA*, *silR*, and *silP*; several *pco* genes also ranked above *merR*, *merP*, and *merA* For sulfisoxazole-resistant isolates, *golS*, *golT*, and *asr* were followed by *merR*, *merP*, and *merA*, with *qacEdelta1* and *merT* also among the higher-ranked genes. *sil* genes occupied the middle portion of the profile, while *terW*, *terD*, *terZ*, *arsD*, and several *pco* genes appeared lower. Ampicillin- and ceftriaxone-resistant isolates showed similar upper-ranking profiles, with *golS*, *golT*, and *asr* followed by *merR*, *merP*, and *merA*. Their lower-ranking profiles included multiple *sil* and *pco* genes, with *ter* genes among the least frequent displayed. In nalidixic acid-resistant isolates, *golS*, *golT*, and *asr* again ranked first through third, followed by *arsR*. *merR*, *merT*, *merC*, *merP*, *qacEdelta1*, *merA*, *merD*, and *merE* were also frequently detected, while *qacE*, *arsD*, and several *sil* genes ranked lower. Chloramphenicol-resistant isolates showed the same upper and middle ordering as tetracycline-resistant isolates for *golS*, *golT*, *asr*, *merR*, *merP*, *merA*, *arsD*, *qacEdelta1*, *merT*, *arsR*, *merC*, *merD*, and *merE*, followed by *sil* and *pco* genes at lower ranks.

### 3.2. Predictive Performance of Logistic Regression Models Based on AMR, Virulence, and Stress-Response Genes Across the Seven Selected Antimicrobials for Resistance

Logistic regression models were evaluated for each of the seven selected antimicrobials using five predictor sets: virulence genes only, stress-response genes only, combined virulence and stress-response genes, AMR genes only, and all three gene categories combined. Model performance was summarized using the area under the receiver operating characteristic curve (AUC). Table 6 integrates the AUC values for all model types and antimicrobials to allow direct comparison of predictive performance across gene categories and resistance phenotypes.

Across the seven antimicrobials, the AMR-only models consistently showed high predictive performance, with AUC values ranging from 0.926 to 0.995. The lowest AMR-only AUC was observed for streptomycin (0.926), whereas tetracycline and nalidixic acid had the highest values (0.995 each). The remaining AMR-only AUC values were 0.993 for sulfisoxazole, 0.993 for ampicillin, 0.991 for ceftriaxone, and 0.993 for chloramphenicol.

In contrast, the virulence-only and stress-response-only models showed greater variation among antimicrobials. Virulence-only AUC values ranged from 0.808 to 0.961, with the highest values observed for sulfisoxazole (0.961) and chloramphenicol (0.948), and the lowest for ampicillin (0.808). Stress-response-only AUC values ranged from 0.775 to 0.987, with the highest values for sulfisoxazole (0.987), chloramphenicol (0.960), and nalidixic acid (0.949), and lower values for ampicillin (0.805), streptomycin (0.788), tetracycline (0.783), and ceftriaxone (0.775).

Combining virulence and stress-response genes increased AUC relative to the better-performing of the two individual non-AMR models for all seven antimicrobials. The increase ranged from 0.003 for sulfisoxazole to 0.105 for tetracycline. The largest increases were observed for tetracycline (+0.105), streptomycin (+0.039), and nalidixic acid (+0.037), followed by ampicillin (+0.018), chloramphenicol (+0.010), ceftriaxone (+0.006), and sulfisoxazole (+0.003). The resulting virulence + stress-response AUC values ranged from 0.826 for ampicillin to 0.990 for sulfisoxazole, with relatively high values also observed for nalidixic acid (0.986), chloramphenicol (0.970), and tetracycline (0.948).

The model containing AMR, virulence, and stress-response genes together produced the highest AUC for each antimicrobial. However, the improvement over the corresponding AMR-only model was relatively small, ranging from 0.001 to 0.009. The increase was 0.001 for tetracycline, 0.009 for streptomycin, 0.003 for sulfisoxazole, 0.003 for ampicillin, 0.006 for ceftriaxone, 0.002 for nalidixic acid, and 0.005 for chloramphenicol. The full-model AUC values were therefore 0.996 for tetracycline, 0.935 for streptomycin, 0.996 for sulfisoxazole, 0.996 for ampicillin, 0.997 for ceftriaxone, 0.997 for nalidixic acid, and 0.998 for chloramphenicol.

Overall, the integrated results show that the virulence-only, stress-response-only, and combined “virulence + stress-response” models varied substantially across antimicrobials, whereas the AMR-only and full models remained consistently high. The full model produced the highest AUC for all seven antimicrobials, while the magnitude of improvement from adding virulence and stress-response genes to the AMR model differed by antimicrobial.

### 3.3. Genotype–Phenotype Discrepancy and Enriched Virulence and Stress-Response Genes in Antimicrobial-Resistant Salmonella enterica

#### 3.3.1. Genotype–Phenotype Discrepancy Across the Seven Selected Antimicrobials

Genotype–phenotype discrepancy analysis was used to identify phenotypically resistant *Salmonella enterica* isolates that lacked the expected antimicrobial-specific AMR genes. Table 7 summarizes the total number of resistant isolates, the number carrying at least one expected AMR determinant, the number classified as discrepant resistant isolates, the corresponding discrepancy percentage, and the expected AMR genes used for each antimicrobial.

Across the seven antimicrobials, discrepant resistant isolates represented 0.06% to 2.10% of the corresponding resistant populations. Tetracycline had 2392 resistant isolates, including 18 discrepant isolates (0.75%); streptomycin had 1559 resistant isolates, of which 1558 carried at least one of the expected AMR genes *aadA1*, *mdsA*, or *mdsB*, leaving one discrepant isolate (0.06%); sulfisoxazole had 1425 resistant isolates and 15 discrepant isolates (1.05%); ampicillin had 985 resistant isolates and 17 discrepant isolates (1.73%); ceftriaxone had 857 resistant isolates and 18 discrepant isolates (2.10%); nalidixic acid had 598 resistant isolates and six discrepant isolates (1.00%); and chloramphenicol had 316 resistant isolates and five discrepant isolates (1.58%).

The number of discrepant isolates ranged from one for streptomycin to 18 for tetracycline and ceftriaxone. When expressed as a percentage of the resistant population, ceftriaxone had the highest discrepancy percentage (2.10%), followed by ampicillin (1.73%), chloramphenicol (1.58%), sulfisoxazole (1.05%), nalidixic acid (1.00%), tetracycline (0.75%), and streptomycin (0.06%).

#### 3.3.2. Virulence and Stress-Response Genes Enriched in Discrepant Resistant Isolates

The discrepant resistant isolates were compared with resistant isolates carrying the expected AMR determinants using Fisher’s exact test with false discovery rate correction. Table 8 combines the virulence and stress-response genes identified as enriched in the discrepant groups. No virulence or stress-response gene was significantly enriched for streptomycin in the corrected discrepancy analysis. To reduce the length of the integrated table, genes belonging to the same named family are placed in a single family-based row across all seven antimicrobial columns. Genes that do not belong to one of the repeated families shown in the table are consolidated under “Other virulence genes” or “Other stress-response genes.”

For tetracycline, the enriched set contained several individual virulence genes, the spv family genes *spvB* and *spvD*, and seven pco stress-response genes. For streptomycin, no virulence or stress-response gene was significantly enriched in the single discrepant resistant isolate; therefore, no enriched gene is listed for streptomycin in Table 8.

For sulfisoxazole, the enriched set included individual virulence genes together with iuc/iut, iro, cva, pco, sil, and ars family genes. Ampicillin contained two enriched virulence genes, *steB* and *stkA*. Ceftriaxone included several individual virulence genes together with the spv family genes *spvB* and *spvD*. Nalidixic acid contained the ybt family genes *ybtQ* and *ybtP* and the stress-response gene *qacEdelta1*. Chloramphenicol contained only stress-response genes in the reported set, represented by pco and sil family groups.

Across the six antimicrobial groups in which enriched genes were identified, several genes or families occurred in more than one group. The pco family was enriched in tetracycline-, sulfisoxazole-, and chloramphenicol-discrepant isolates, while the sil family was enriched in sulfisoxazole- and chloramphenicol-discrepant isolates. The spv family was enriched in tetracycline- and ceftriaxone-discrepant isolates. Among individual genes, *steB* was identified for tetracycline, sulfisoxazole, ampicillin, and ceftriaxone, whereas *stkA* was identified for sulfisoxazole and ampicillin.

## 4. Discussion

### 4.1. Antimicrobial-Specific Resistance Determinants Are Embedded Within a Shared Multidrug Genomic Background

The present study provides an integrated view of antimicrobial-resistant *Salmonella enterica* from chicken sources by examining canonical AMR genes together with virulence and stress-response genes across seven antimicrobial phenotypes. The main contribution is not simply the observation that these gene categories can co-occur; such genomic linkages have been recognized previously. Rather, this study combines antimicrobial-specific gene-occurrence profiling, direct comparison of the predictive performance of AMR, virulence, and stress-response gene sets, and targeted analysis of genotype–phenotype discrepant isolates within the same large chicken-associated dataset. This framework makes it possible to distinguish the expected antimicrobial-specific resistance signal from the broader adaptive genomic background in which resistance occurs.

The AMR-gene rankings provide an important internal validation of the analytical approach. Genes with well-established relevance to the corresponding antimicrobial rose to prominent positions in the appropriate resistant groups: *tet(A)* and *tet(B)* were highly ranked for tetracycline; *aph(3″)-Ib*, *aph(6)-Id*, and *aadA1* were prominent for streptomycin; *sul1* and *sul2* were prominent for sulfisoxazole; *bla_CMY-2_* and *bla_CTX-M-65_* were prominent in the beta-lactam groups; *gyrA_D87Y* was especially prominent for nalidixic acid; and *floR* was highly ranked for chloramphenicol. This agreement with known resistance biology is consistent with previous studies showing strong correspondence between whole-genome resistance determinants and phenotypic antimicrobial susceptibility in nontyphoidal *Salmonella* [15,22,62]. It also supports the interpretation that the cross-antimicrobial differences observed for virulence and stress-response genes are being detected against a biologically credible AMR background rather than arising solely from an arbitrary gene-selection procedure.

At the same time, the resistant isolates were not characterized only by antimicrobial-specific determinants. *mdsA* and *mdsB* ranked first and second in every resistant group, and *tet(A)*, *sul* genes, aminoglycoside-resistance genes, beta-lactamase genes, and other determinants frequently appeared in groups defined by resistance to different antimicrobial classes. This broad overlap is consistent with reports that *Salmonella* from food and animal sources can carry multiple AMR genes and multidrug-resistance profiles [25]. The repeated detection of *mdsA* and *mdsB* is also biologically plausible because MdsABC is a *Salmonella*-specific tripartite efflux system with functions extending beyond a single drug phenotype; experimental work has linked MdsABC to responses to toxic compounds, oxidative stress, survival in phagocytic cells, and virulence-associated phenotypes [63]. Thus, the frequency rankings are best interpreted as showing that antimicrobial-specific determinants are embedded within a shared multidrug and adaptive genomic background.

An additional consideration in interpreting the shared genomic background is that the seven antimicrobial-resistant groups were not mutually exclusive. Individual *Salmonella enterica* isolates could be resistant to more than one antimicrobial and therefore could contribute to more than one antimicrobial-specific analysis. This study intentionally analyzed each resistance phenotype separately because the expected AMR determinants, resistance outcome, and genotype–phenotype discrepancy definition differ by antimicrobial. Thus, for logistic regression, each model evaluated resistance to one antimicrobial as the outcome, and for discrepancy analysis, resistant isolates were evaluated specifically for the expected determinants of that antimicrobial. The repeated occurrence of genes such as *mdsA*, *mdsB*, *tet(A)*, *sul* genes, beta-lactamase genes, and aminoglycoside-resistance genes across multiple antimicrobial groups should therefore not be interpreted as independent replication across seven distinct isolate populations. Rather, part of this overlap likely reflects multidrug-resistant isolates carrying determinants for several antimicrobial classes. This pattern is itself consistent with previous reports of multidrug-resistant *Salmonella* from poultry and food-animal sources and with mobile genetic elements that carry multiple resistance [20,27,64]. The antimicrobial-specific design nevertheless allows the genomic background associated with each resistance phenotype to be characterized separately while preserving the biological reality that multidrug resistance frequently occurs within the same isolate.

This broader background is particularly relevant to poultry-associated *Salmonella* because clinically important resistance determinants can occur on mobile elements that also carry other adaptive traits. For example, multidrug-resistant *Salmonella Infantis* lineages carrying *bla_CTX-M-65_* have been recovered from humans and retail chicken in the United States, and pESI-like plasmids in *Salmonella Infantis* can harbor resistance determinants to multiple antimicrobial classes together with virulence-associated loci [19,65]. These observations provide a plausible genomic basis for the extensive cross-class overlap seen in the present AMR profiles and reinforce the need to interpret resistant isolates as genomic backgrounds rather than as collections of isolated resistance genes.

### 4.2. Virulence and Stress-Response Genes Capture Antimicrobial-Resistance-Associated Genomic Backgrounds

A major finding of this study is that virulence and stress-response genes contained substantial information for distinguishing resistant from susceptible isolates even when canonical AMR genes were excluded from the model. The magnitude of this signal was antimicrobial dependent. The combined virulence + stress-response model reached AUC values of 0.990 for sulfisoxazole, 0.986 for nalidixic acid, 0.970 for chloramphenicol, and 0.948 for tetracycline. Stress-response genes alone were especially informative for sulfisoxazole, chloramphenicol, and nalidixic acid, whereas virulence genes alone were particularly informative for sulfisoxazole and chloramphenicol. These results extend the descriptive gene-frequency findings by showing that non-AMR genomic features are not merely present in resistant isolates; for several resistance phenotypes, their combined occurrence patterns strongly classify the resistance-associated genomic background.

The corresponding AMR-only models nevertheless remained the strongest individual-category models for all seven antimicrobials, with AUC values of 0.926–0.995. Adding virulence and stress-response genes to the AMR models increased AUC by only 0.001–0.009. This pattern is important because it places the non-AMR findings in the correct biological context. The virulence and stress-response genes do not replace canonical AMR determinants as the primary predictors of resistance. Instead, they capture additional structure that accompanies resistance. The high performance of the non-AMR models may reflect co-selection, shared mobile genetic elements, clonal or serovar-associated genomic backgrounds, or ecological selection for traits that promote persistence under multiple stresses. Accordingly, predictive association should not be interpreted as evidence that a virulence or stress-response gene directly confers resistance to the antimicrobial.

The presence of multidrug-resistant isolates also provides an important context for these predictive associations. Because an isolate resistant to several antimicrobials could be included in the separate model developed for each corresponding phenotype, a virulence or stress-response gene associated with a successful multidrug-resistant lineage may contribute predictive information for more than one antimicrobial. The antimicrobial-specific logistic-regression models therefore estimate how well each gene category distinguishes the resistance phenotype under study; they do not establish that a non-AMR gene is uniquely associated with that antimicrobial or independent of resistance to other drugs. This distinction is particularly important when interpreting the strong virulence- and stress-response-only models for sulfisoxazole, nalidixic acid, and chloramphenicol. Their high AUC values may partly reflect broader multidrug-resistant genomic backgrounds in addition to features associated with the individual resistance phenotype.

This interpretation is supported by previous genomic studies. AMRFinderPlus was explicitly expanded to include stress-response and virulence genes so that genomic linkages among AMR, stress adaptation, and virulence could be examined in foodborne pathogens [21,64]. Analyses of NCBI Pathogen Detection data have also reported recurrent stress-response genes, including *asr*, *ars*, *mer*, *qacEdelta1*, *golS*, and *golT*, in antimicrobial-resistant pathogens and have identified concurrent relationships among stress-response, AMR, and virulence genes in *Salmonella* [29,30]. Similarly, virulence genes such as *iss*, *iutA*, *ybtP*, and *ybtQ* have been detected in antimicrobial-resistant foodborne-pathogen datasets [36]. The recurrence of *golS*, *golT*, and *asr* at the top of all seven stress-response profiles in the present study, together with the repeated detection of *mer*, *ars*, *pco*, and *sil* systems, is therefore consistent with an adaptive stress background that has been observed independently in large genomic datasets.

The predictive results also align with recent work showing that genome-scale models can predict antimicrobial resistance in *Salmonella* isolated from chicken with high accuracy [66]. The present study adds a different dimension by separating genes into mechanistically distinct categories rather than treating the genome only as a pool of predictive features. This comparison shows that models based on annotated AMR genes provided the highest AUC values among the individual-category models, whereas models based on virulence or stress-response genes also distinguished resistant from susceptible isolates for selected antimicrobials. These findings describe the predictive performance of gene sets; they do not establish that individual virulence or stress-response genes independently predict resistance or directly confer it. A model may achieve high predictive performance because its predictors reflect shared lineages or genomic backgrounds rather than direct resistance mechanisms. The present study deals with the observed co-occurrence and statistical associations of AMR, virulence, and stress-response genes within the analyzed isolates. These associations do not establish that the non-AMR genes directly mediate antimicrobial resistance or that they are physically linked to AMR determinants in the analyzed isolates. The observed patterns may reflect shared genomic backgrounds, co-selection, or other factors that cannot be distinguished by the present analysis alone. Additional genomic and experimental studies are needed to determine which, if any, of the identified genes contribute functionally to the observed resistance phenotypes.

### 4.3. Genotype–Phenotype Discrepancy Identifies Rare but Informative Adaptive Genomic Signatures

The discrepancy analysis provides a second major contribution of this study. Across the seven antimicrobials, only 0.06–2.10% of resistant isolates lacked the expected antimicrobial-specific AMR determinants. The rarity of these cases is consistent with the generally high genotype–phenotype concordance reported for *Salmonella* WGS. Neuert et al., 2018 [22], reported very high agreement between WGS-derived genotypic profiles and phenotypic susceptibility in nontyphoidal *Salmonella*, and McDermott et al., 2016 [15], and Cooper et al., 2020 [23], similarly demonstrated that known genomic resistance determinants predict most phenotypic resistance. Thus, the low discrepancy frequencies in this study strengthen confidence in the expected-AMR definitions while identifying a small set of exceptions that warrant focused investigation.

The enrichment results should be interpreted cautiously because the number of genotype–phenotype discrepant isolates was small for several antimicrobials, particularly streptomycin (*n* = 1), chloramphenicol (*n* = 5), and nalidixic acid (*n* = 6). Although Fisher’s exact test is appropriate for analyses involving small cell counts, the limited number of discrepant isolates constrains the strength and stability of the resulting gene-association estimates. Consequently, genes identified as enriched in these small groups should be regarded as exploratory candidates associated with the genomic backgrounds of the discrepant isolates rather than as established markers of unexplained antimicrobial resistance. These associations require confirmation in larger, independent isolate populations before broader conclusions can be drawn. The biological value of these exceptions is illustrated by the enrichment patterns. The *pco* family was enriched in discrepant isolates for tetracycline, sulfisoxazole, and chloramphenicol, while *sil* genes were enriched for sulfisoxazole and chloramphenicol. These are not conventional determinants of resistance to the corresponding antibiotics, but their recurrence is biologically plausible in a resistance-associated genomic background. A large analysis of 56,348 *Salmonella* genomes documented co-occurrence between antibiotic and metal-resistance determinants and highlighted *pco*/*sil*-associated metal-resistance patterns in multidrug-resistant populations [67]. More directly, experimental work has demonstrated that the acquired *pco* cluster in *Salmonella enterica* mediates copper resistance [68]. These studies support interpretation of the *pco* and *sil* enrichments as markers of metal-stress adaptation and possible co-selection rather than as evidence that these genes directly cause tetracycline, sulfisoxazole, or chloramphenicol resistance.

The nalidixic acid discrepancy profile provides another example. *qacEdelta1*, a stress-response/biocide-associated gene, was enriched together with the virulence-associated *ybtQ* and *ybtP* genes. Experimental and genomic work has shown that *qacEΔ1* in class 1 integrons can confer disinfectant resistance and occur in genetic contexts associated with antibiotic-resistance determinants in *Salmonella* [69]. The *ybt* locus encodes the yersiniabactin iron-acquisition system, and pESI-like *Salmonella Infantis* megaplasmids provide a precedent for physical linkage of antimicrobial-resistance determinants with virulence and iron-acquisition traits, including yersiniabactin-associated loci [65,70]. Therefore, enrichment of *qacEdelta1* and *ybtP*/*ybtQ* in discrepant nalidixic acid-resistant isolates is consistent with the broader concept that unusual resistant isolates may be embedded in mobile-element or lineage backgrounds carrying multiple adaptive functions.

Virulence genes also recurred across several discrepant groups. *steB* was enriched for tetracycline, sulfisoxazole, ampicillin, and ceftriaxone, while *stkA* was enriched for sulfisoxazole and ampicillin; *spv* genes were enriched in the tetracycline and ceftriaxone groups. These repeated signals should be treated as candidate markers of particular genomic backgrounds rather than as alternative resistance genes. Their recurrence is nevertheless noteworthy because the comparison was restricted to resistant isolates: discrepant resistant isolates were compared with resistant isolates already carrying expected AMR genes. Consequently, these enrichments identify features associated specifically with the unexplained subset rather than simply distinguishing resistant from susceptible populations.

The streptomycin result further illustrates the limitations of enrichment analysis when the discrepant group is extremely small. Only one of 1559 streptomycin-resistant isolates was discrepant, and no virulence or stress-response gene was significantly enriched. Given the presence of only one discrepant isolate, this result should not be interpreted as evidence that no virulence or stress-response genes are associated with unexplained streptomycin resistance.

### 4.4. Significance for Genomic Surveillance and One Health

Together, the logistic-regression and discrepancy results suggest that genomic surveillance of antimicrobial-resistant *Salmonella* can benefit from retaining information beyond canonical AMR determinants. Models based on annotated AMR genes showed the most consistent predictive performance, whereas virulence and stress-response gene profiles provided additional information about resistance-associated genomic backgrounds. This is conceptually consistent with the design of the NCBI Reference Gene Catalog and AMRFinderPlus, which includes stress-response and virulence genes specifically to facilitate examination of genomic linkages among these functions [21]. The present analysis shows why that broader annotation can be useful: it identifies shared adaptive signatures across resistant populations and highlights unusual resistant isolates whose expected resistance determinants are absent.

For food-chain surveillance, this broader context may be particularly relevant to persistence and dissemination. Metal-resistance systems, biocide-associated genes, virulence loci, and AMR determinants can coexist on the same mobile elements or within successful clonal backgrounds. The pESI/pESI-like *Salmonella Infantis* lineage provides a well-characterized example in which antimicrobial resistance, stress tolerance, and pathogenicity-associated features occur together and contribute to the success of an emergent poultry-associated lineage [19,65,70]. The present study does not establish that the enriched genes are physically linked to AMR determinants in the analyzed isolates, but it identifies specific gene families and antimicrobial phenotypes for which such linkage should be tested.

The practical implication is not that virulence or stress-response markers should replace antimicrobial susceptibility testing or established AMR-gene prediction. Rather, they may provide additional information for identifying adaptive genomic backgrounds and interpreting rare genotype–phenotype discrepancies. This perspective is relevant to One Health surveillance because chicken-associated *Salmonella* circulates among animal, food, environmental, and human-associated niches exposed to multiple selective pressures. Similar patterns have been reported previously: metal-resistance genes in *Salmonella* have been associated with antimicrobial and disinfectant resistance [71], and a genomic analysis of 56,348 *Salmonella* isolates identified co-occurrence of antimicrobial- and metal-resistance determinants, including *pco* and *sil* systems [67]. In addition, One Health analyses of *Salmonella Infantis* have linked multidrug resistance and pESI-like plasmids with successful poultry-associated lineages across human, animal, and food sources [72]. These reports are consistent with our findings, although further studies are needed to determine whether these associations reflect co-selection, genetic linkage, or shared clonal backgrounds.

### 4.5. Limitations and Future Work

Several limitations should be considered when interpreting these findings. First, the downstream analyses initially used genes prioritized through a sequential screening procedure integrating PCA, hierarchical clustering, and frequency-based bar-plot analysis. Although this procedure allowed us to identify genes exhibiting distinct or outlying occurrence patterns and relatively high frequencies among resistant isolates, the identification of PCA outliers, distinct clustering patterns, and elbow points involved visual assessment rather than universally defined numerical thresholds. Consequently, the selection of individual candidate genes may depend partly on the exploratory screening criteria. To evaluate whether the main findings depended on this initial gene-prioritization procedure, we repeated the logistic-regression and discrepancy analyses using all genes in the corresponding AMR, virulence, and stress-response categories. The overall AUC patterns and the discrepancy/enrichment conclusions did not materially change, suggesting that the principal findings were not strongly dependent on the initial gene-selection procedure. Nevertheless, the prioritization of individual candidate genes remains exploratory and warrants further validation.

An additional limitation is the small number of genotype–phenotype discrepant isolates for several antimicrobials. In particular, only one, five, and six discrepant isolates were identified for streptomycin, chloramphenicol, and nalidixic acid, respectively. Although Fisher’s exact test was selected because it accommodates small contingency-table cell counts, the limited number of discrepant isolates restricts the precision and generalizability of the enrichment results. The enriched genes identified in these small groups should therefore be interpreted as preliminary associations rather than definitive resistance-associated markers. Similarly, the absence of statistically significant enrichment for streptomycin does not exclude potentially relevant associations. Future studies using larger collections of discrepant isolates are needed to validate the identified genes and assess the reproducibility of these enrichment patterns.

In addition, the dataset used for the genotype–phenotype discrepancy analysis included only phenotypically resistant isolates. Therefore, the opposite type of genotype–phenotype discrepancy, in which an expected AMR determinant is detected in a susceptible isolate, could not be investigated. Future studies incorporating both resistant and susceptible isolates will be needed to examine this complementary form of discordance.

As another limitation, enrichment testing in the discrepancy analysis was intentionally focused on virulence and stress-response genes rather than AMR genes. This choice reflected the primary research question: whether resistant isolates lacking the expected antimicrobial-specific AMR determinants carry a distinctive non-AMR adaptive background. It should not be interpreted to mean that alternative AMR mechanisms were excluded biologically. Future work should systematically examine discrepant isolates for additional acquired AMR genes, resistance-associated point mutations, structural variants, promoter or regulatory changes, altered efflux regulation, gene copy-number differences, and other mechanisms not captured by the expected-gene lists. Heteroresistance is another possibility because phenotypically resistant subpopulations can be missed by conventional WGS analysis of the dominant population [73].

Another limitation is that resistance to the seven antimicrobials was analyzed as separate antimicrobial-specific phenotypes even though individual isolates could exhibit resistance to multiple antimicrobials. Consequently, the resistant groups were overlapping rather than mutually exclusive, and the same multidrug-resistant isolate could contribute to the analysis of more than one antimicrobial. This design was intentional because both logistic regression and genotype–phenotype discrepancy were defined separately for each antimicrobial. Within an individual logistic-regression analysis, each isolate was evaluated only once for the corresponding resistance outcome, and within an individual discrepancy analysis, resistant isolates were classified according to the expected AMR determinants for that specific antimicrobial. Therefore, multidrug resistance does not create duplicate observations within a given antimicrobial-specific model. However, it means that comparisons of gene patterns across the seven antimicrobials should not be interpreted as comparisons of seven statistically independent isolate populations. Shared AMR, virulence, and stress-response patterns may partly reflect multidrug-resistant lineages, co-selection, or linked resistance determinants rather than independent associations with each individual antimicrobial.

Finally, the present study is computational and does not demonstrate that enriched virulence or stress-response genes mechanistically facilitate antibiotic resistance. Future work should combine long-read or plasmid-resolved sequencing with phylogenetic analysis to determine whether AMR, metal-resistance, biocide-resistance, and virulence genes are physically linked on the same mobile elements. Transcriptomic analysis could determine whether candidate stress-response systems are activated during antibiotic exposure, while phenotypic assays could test metal and disinfectant tolerance in discrepant versus expected-AMR isolates. Experimental perturbation of prioritized genes or operons would be required before assigning a direct functional role in antimicrobial resistance. External validation of the logistic-regression models, ideally with temporally or geographically independent chicken-associated *Salmonella* isolates, would further establish the generalizability of the predictive patterns.

## 5. Conclusions

This study shows that antimicrobial-resistant *Salmonella enterica* from chicken sources is characterized by both antimicrobial-specific resistance determinants and a broader adaptive genomic background. The expected AMR signals were recovered across all seven antimicrobials, and AMR-only logistic-regression models consistently provided the strongest individual-category prediction. At the same time, virulence and stress-response genes exhibited recurring occurrence patterns across antimicrobial groups, and models based on these gene categories distinguished resistant from susceptible isolates with high AUC values for several phenotypes, even without AMR-gene predictors. The antimicrobial-specific analyses also revealed substantial overlap among resistance-associated gene profiles, consistent with the presence of isolates carrying resistance to multiple antimicrobial classes; accordingly, these shared patterns represent overlapping multidrug-resistant genomic backgrounds rather than seven completely independent resistant populations. Genotype–phenotype discrepancy was uncommon, occurring in 0.06–2.10% of resistant isolates, but these rare cases provided additional information that would be missed by analysis of canonical AMR genes alone. Discrepant isolates for several antimicrobials were enriched for stress-response systems associated with metal or biocide tolerance, including *pco*, *sil*, and *qacEdelta1*, as well as selected virulence-associated genes and gene families. These enrichments do not establish direct alternative resistance mechanisms, but they identify adaptive genomic backgrounds that can now be tested for co-selection, mobile-element linkage, regulatory effects, or previously unrecognized resistance mechanisms. Therefore, the present findings should be interpreted as hypothesis-generating genomic associations rather than evidence that the identified virulence or stress-response genes directly contribute to antimicrobial resistance. Experimental and phylogenetically controlled validation will be essential to determine which of these associations are markers of shared genomic background and which, if any, contribute functionally to resistance or persistence.

## Figures and Tables

**Table 1:** Expected antimicrobial-resistance determinants used in the genotype–phenotype discrepancy analysis.

Antimicrobial	Expected AMR Determinants
Tetracycline	*tet(A)*, *tet(B)*, *tet(C)*, *tet(G)*
Streptomycin	*mdsA*, *mdsB*, *aadA1*, *aph(3*″*)-Ib*, *aph(6)-Id*
Sulfisoxazole	*sul1*, *sul2*, *sul3*
Ampicillin	*blaCARB*, *blaCMY*, *blaCTX*, *blaHER*, *blaTEM*
Ceftriaxone	*blaCTX-M*, *blaCTX-M-1*, *blaCTX-M-65*, *blaCMY*, *blaCMY-2*
Nalidixic acid	*qnrA*, *qnrS*, *qnrB*, *gyrA_D82N*, *gyrA_D87Y*, *gyrA_S83F*, *qnrB19*
Chloramphenicol	*floR*, *cat*, *cmlA*, *fex*, *optrA*, *poxtA*

**Table 2:** Contingency table for Fisher’s exact test comparing gene presence and absence between discrepant resistant isolates and resistant isolates carrying expected AMR genes.

	Gene Present	Gene Absent
Discrepant resistant isolates	*a*	*b*
Resistant isolates with expected AMR genes	*c*	*d*

**Table 3:** Comparative ranking of AMR genes by occurrence among *Salmonella enterica* isolates resistant to the seven selected antimicrobials.

Rank	Tetracycline	Streptomycin	Sulfisoxazole	Ampicillin	Ceftriaxone	Nalidixic Acid	Chloramphenicol
1	*mdsA*	*mdsA*	*mdsA*	*mdsA*	*mdsA*	*mdsA*	*mdsA*
2	*mdsB*	*mdsB*	*mdsB*	*mdsB*	*mdsB*	*mdsB*	*mdsB*
3	*tet(A)*	*aph(3* *″* *)-Ib*	*tet(A)*	*tet(A)*	*tet(A)*	*gyrA_D87Y*	*floR*
4	*aph(3* *″* *)-Ib*	*aph(6)-Id*	*sul2*	*bla_CMY-2_*	*bla_CMY-2_*	*sul1*	*gyrA_D87Y*
5	*aph(6)-Id*	*tet(B)*	*sul1*	*sul2*	*sul2*	*tet(A)*	*tet(A)*
6	*tet(B)*	*tet(A)*	*aadA1*	*sul1*	*gyrA_D87Y*	*aadA1*	*aph(4)-Ia*
7	*sul2*	*aadA1*	*gyrA_D87Y*	*gyrA_D87Y*	*bla_CTX-M-65_*	*aac(3)-IVa*	*aac(3)-IVa*
8	*sul1*	*sul1*	*bla_CMY-2_*	*bla_CTX-M-65_*	*sul1*	*aph(4)-Ia*	*sul1*
9	*aadA1*	*gyrA_D87Y*	*aph(3′)-Ia*	*aadA1*	*aadA1*	*floR*	*aadA1*
10	*gyrA_D87Y*	*aac(3)-IVa*	*aac(3)-IVa*	*aph(3′)-Ia*	*aph(3′)-Ia*	*bla_CTX-M-65_*	*bla_CTX-M-65_*
11	*bla_CMY-2_*	*aph(4)-Ia*	*aph(4)-Ia*	*aph(4)-Ia*	*aph(4)-Ia*	*dfrA14*	*dfrA14*
12	*aph(3′)-Ia*	*floR*	*floR*	*aac(3)-IVa*	*aac(3)-IVa*	*aph(3′)-Ia*	*aph(3′)-Ia*
13	*aac(3)-IVa*	*bla_CMY-2_*	*bla_CTX-M-65_*	*floR*	*floR*	*fosA3*	*fosA3*
14	*aph(4)-Ia*	*aph(3′)-Ia*	*dfrA14*	*aph(3* *″* *)-Ib*	*aph(3* *″* *)-Ib*	*bla_CTX-M_*	*sul2*
15	*floR*	*bla_CTX-M-65_*	*aph(3* *″* *)-Ib*	*aph(6)-Id*	*aph(6)-Id*	*bla_TEM-1_*	*aph(3* *″* *)-Ib*
16	*bla_CTX-M-65_*	*sul2*	*aac(3)-VIa*	*bla_TEM-1_*	*dfrA14*	*aadA2*	*aph(6)-Id*
17	*dfrA14*	*dfrA14*	*bla_TEM-1_*	*dfrA14*	*tet(B)*	*gyrA_S83F*	*bla_CTX-M_*
18	*bla_TEM-1_*	*aac(3)-VIa*	*aph(6)-Id*	*tet(B)*	*fosA3*	*ant(2* *″* *)-Ia*	*bla_CMY-2_*
19	*aac(3)-VIa*	*fosA7*	*fosA3*	*fosA3*	*bla_TEM-1_*	*aph(3* *″* *)-Ib*	*bla_TEM-1_*
20	*fosA3*	*fosA3*	*fosA7*	*bla_CMY_*	*bla_CMY_*	*aph(6)-Id*	*ant(2* *″* *)-Ia*
21	*fosA7*	*bla_TEM-1_*	*tet(C)*	*fosA7*	*fosA7*		*cmlA5*
22	*bla_CMY_*	*tet(C)*	*bla_CMY_*	*aac(3)-VIa*	*bla_CTX-M_*		*fosA7*
23	*tet(C)*	*aph(3′)-IIa*	*tet(B)*	*ant(2* *″* *)-Ia*	*dfrA51*		*aadA2*
24	*dfrA51*	*aph(6)-Ic*	*dfrA51*	*bla_CTX-M_*	*aac(3)-VIa*		*bla_CARB-2_*
25	*aph(3′)-IIa*	*ble*	*bla_CTX-M_*	*dfrA51*	*ant(2* *″* *)-Ia*		*tet(G)*
26		*bla_CMY_*	*ant(2* *″* *)-Ia*	*aadA2*	*cmlA5*		*aac(3)-VIa*
27		*ant(2* *″* *)-Ia*	*aadA2*	*cmlA5*	*bla_TEM_*		*bla_TEM_*
28		*bla_CTX-M_*	*cmlA5*	*bla_TEM_*	*aph(3′)-IIa*		*bleO*
29		*aadA2*	*bla_TEM_*	*bla_CARB-2_*	*aph(6)-Ic*		*dfrA12*
30		*cmlA5*	*blaCARB-2*	*tet(G)*	*ble*		*dfrA51*

Note: Within each antimicrobial column, genes are ordered from highest to lowest occurrence among resistant isolates. Ranks are specific to each antimicrobial and should not be interpreted as direct comparisons of absolute prevalence between antimicrobial groups.

**Table 4:** Comparative ranking of virulence genes by occurrence in *Salmonella enterica* isolates resistant to the seven selected antimicrobials.

Rank	Tetracycline	Streptomycin	Sulfisoxazole	Ampicillin	Ceftriaxone	Nalidixic Acid	Chloramphenicol
1	*invA*	*invA*	*iroB*	*iroB*	*iroB*	*iroB*	*avrA*
2	*iroB*	*iroC*	*sinH*	*sinH*	*sinH*	*sinH*	*fljA*
3	*iroC*	*sinH*	*invA*	*avrA*	*avrA*	*avrA*	*hscC*
4	*sinH*	*iroB*	*iroC*	*invA*	*invA*	*invA*	*icmF*
5	*pipA*	*pipA*	*lsrF*	*iroC*	*iroC*	*iroC*	*invA*
6	*avrA*	*icmF*	*icmF*	*pipA*	*lsrF*	*lsrF*	*iroB*
7	*hscC*	*avrA*	*pipA*	*lsrF*	*pipA*	*pipA*	*iroC*
8	*envF*	*lsrF*	*avrA*	*hscC*	*allD*	*allD*	*lsrF*
9	*stfA*	*sciR*	*sciR*	*stfA*	*gip*	*gip*	*pipA*
10	*allD*	*fljA*	*allD*	*envF*	*hscC*	*hscC*	*sinH*
11	*icmF*	*allD*	*gip*	*lpfB*	*stfA*	*stfA*	*stfA*
12	*gip*	*hscC*	*hscC*	*allD*	*envF*	*envF*	*lpfB*
13	*lpfB*	*gip*	*stfA*	*gip*	*lpfB*	*lpfB*	*sciR*
14	*sciR*	*stfA*	*envF*	*icmF*	*icmF*	*icmF*	*envF*
15	*lsrF*	*envF*	*lpfB*	*sciR*	*fljA*	*fljA*	*ripR*
16	*fljA*	*lpfB*	*fljA*	*fljA*	*sciR*	*sciR*	*sspH2*
17	*stiA*	*stiA*	*ripR*	*stiA*	*stiA*	*stiA*	*stm0859*
18	*stjB*	*stjB*	*stm0859*	*ripR*	*ripR*	*ripR*	*allD*
19	*stcA*	*stcA*	*pagO*	*stm0859*	*stm0859*	*stm0859*	*gip*
20	*stcD*	*stcD*	*stm0854*	*pagO*	*pagO*	*pagO*	*stm0854*
21	*yigF*	*yigF*	*stiA*	*stm0854*	*stm0854*	*stm0854*	*pagO*
22	*sseK2*	*stkA*	*sseK2*	*sspH2*	*sseK2*	*sseK2*	*sseK2*
23	*ripR*	*steB*	*sspH2*	*sseK2*	*sspH2*	*sspH2*	*pagK*
24	*stm0859*	*iucA*	*pagK*	*pagK*	*pagK*	*pagK*	*stiA*
25	*pagO*	*iucB*	*safB*	*safB*	*safB*	*safB*	*safB*
26			*ybtQ*	*stjB*	*ybtQ*	*ybtQ*	*tcfA*
27			*ybtP*	*stcA*	*ybtP*	*ybtP*	*tinR*
28			*stjB*	*stcD*	*stjB*	*stjB*	*ybtQ*
29			*stcA*	*ybtQ*	*stcA*	*stcA*	*ybtP*
30			*stcD*	*ybtP*	*stcD*	*stcD*	*stjB*

**Table 5:** Comparative ranking of stress-response genes by occurrence in *Salmonella enterica* isolates resistant to the seven selected antimicrobials.

Rank	Tetracycline	Streptomycin	Sulfisoxazole	Ampicillin	Ceftriaxone	Nalidixic Acid	Chloramphenicol
1	*golS*	*golS*	*golS*	*golS*	*golS*	*golS*	*golS*
2	*golT*	*golT*	*golT*	*golT*	*golT*	*golT*	*golT*
3	*asr*	*asr*	*asr*	*asr*	*asr*	*asr*	*asr*
4	*merR*	*arsD*	*merR*	*merR*	*merR*	*arsR*	*merR*
5	*merP*	*silE*	*merP*	*merP*	*merP*	*merR*	*merP*
6	*merA*	*silB*	*merA*	*merA*	*merA*	*merT*	*merA*
7	*arsD*	*silC*	*qacEdelta1*	*arsR*	*arsR*	*merC*	*arsD*
8	*qacEdelta1*	*silF*	*merT*	*qacEdelta1*	*merT*	*merP*	*qacEdelta1*
9	*merT*	*silS*	*merC*	*merT*	*qacEdelta1*	*qacEdelta1*	*merT*
10	*arsR*	*silA*	*merD*	*merD*	*merD*	*merA*	*arsR*
11	*merC*	*silR*	*merE*	*merE*	*merC*	*merD*	*merC*
12	*merD*	*silP*	*arsR*	*merC*	*merE*	*merE*	*merD*
13	*merE*	*pcoS*	*silE*	*arsD*	*arsD*	*qacE*	*merE*
14	*silE*	*pcoC*	*silS*	*silE*	*silE*	*arsD*	*silE*
15	*silB*	*pcoD*	*silA*	*silA*	*pcoA*	*silA*	*silB*
16	*silC*	*pcoR*	*silB*	*silB*	*pcoB*	*silB*	*silC*
17	*silF*	*pcoA*	*silC*	*silC*	*pcoC*	*silC*	*silF*
18	*silA*	*pcoB*	*silF*	*silF*	*pcoD*	*silE*	*silA*
19	*silR*	*merR*	*silP*	*silP*	*pcoR*	*silF*	*silR*
20	*silS*	*merP*	*silR*	*silR*	*pcoS*	*silP*	*silS*
21	*silP*	*merA*	*terW*	*silS*	*silA*		*silP*
22	*pcoS*	*qacEdelta1*	*terD*	*pcoS*	*silB*		*pcoS*
23	*pcoC*	*merT*	*terZ*	*pcoA*	*silC*		*pcoC*
24	*pcoD*	*arsR*	*arsD*	*pcoC*	*silF*		*pcoD*
25	*pcoR*	*merD*	*pcoS*	*pcoD*	*silP*		*pcoR*
26			*pcoA*	*pcoR*	*silR*		
27			*pcoC*	*pcoB*	*silS*		
28			*pcoD*	*pcoE*	*pcoE*		
29			*pcoR*	*terW*	*terD*		
30			*pcoB*	*terD*	*terW*		

**Table 6:** AUC values of logistic regression models based on AMR, virulence, and stress-response genes for resistance to the seven selected antimicrobials.

Model	Tetracycline	Streptomycin	Sulfisoxazole	Ampicillin	Ceftriaxone	Nalidixic Acid	Chloramphenicol
Virulence only	0.843	0.843	0.961	0.808	0.831	0.837	0.948
Stress-response only	0.783	0.788	0.987	0.805	0.775	0.949	0.960
Virulence + Stress-response	0.948	0.882	0.990	0.826	0.837	0.986	0.970
AMR only	0.995	0.926	0.993	0.993	0.991	0.995	0.993
AMR + Virulence + Stress-response	0.996	0.935	0.996	0.996	0.997	0.997	0.998

**Table 7:** Genotype–phenotype discrepancy statistics for *Salmonella enterica* isolates resistant to the seven selected antimicrobials.

Item	Tetracycline	Streptomycin	Sulfisoxazole	Ampicillin	Ceftriaxone	Nalidixic Acid	Chloramphenicol
Total resistant isolates	2392	1559	1425	985	857	598	316
Resistant isolates with expected AMR genes	2374	1558	1410	968	839	592	311
Discrepant resistant isolates	18	1	15	17	18	6	5
Discrepant resistant isolates (%)	0.75	0.06	1.05	1.73	2.10	1.00	1.58

**Table 8:** Virulence and stress-response genes enriched in genotype–phenotype discrepant *Salmonella enterica* isolates across the seven selected antimicrobials, grouped by gene family.

Gene FAMILY/Group	Tetracycline	Streptomycin	Sulfisoxazole	Ampicillin	Ceftriaxone	Nalidixic Acid	Chloramphenicol
Other virulence genes (V)	*bfpA*, *sefA*, *sty2629*, *vasL*, *mltE*, *rcK*, *steB*, *sopE*	None significantly enriched	*steB*, *stkA*, *sty3281*, *iss*, *bfpA*	*steB*, *stkA*	*bfpA*, *sefA*, *sty2629*, *vasL*, *mltE*, *rcK*, *cdtB*, *hlyE*, *pltA*, *steB*		
*spv* family (V)	*spvB*, *spvD*				*spvB*, *spvD*		
iuc/iut genes (V)			*iucA*, *iucB*, *iucC*, *iucD*, *iutA*				
*iro* family (V)			*iroD*, *iroE*, *iroN*				
*cva* family (V)			*cvaB*, *cvaC*				
*ybt* family (V)						*ybtQ*, *ybtP*	
*ars* family (S)			*arsD*				
*pco* family (S)	*pcoE*, *pcoB*, *pcoA*, *pcoC*, *pcoD*, *pcoR*, *pcoS*		*pcoB*, *pcoA*, *pcoC*, *pcoD*, *pcoR*, *pcoS*				*pcoA6*, *pcoB*, *pcoC*, *pcoD*, *pcoR*, *pcoS*
*sil* family (S)			*silA*, *silB*, *silC*, *silF*, *silP*, *silR*, *silE*, *silS*				*silA*, *silB*, *silC*, *silE*, *silF*, *silP*, *silS*
Other stress-response genes (S)						*qacEdelta1*	

Note. (V) = virulence gene; (S) = stress-response gene. The genes listed in this table were identified by individual gene-level enrichment analysis and are grouped by gene family for concise presentation. Their inclusion does not imply that every discrepant isolate carried each listed gene or that all genes within a family occurred together in the same isolates. The total number of discrepant isolates for each antimicrobial is reported in Table 7.

## Data Availability

The data analyzed in this study are publicly available through the NCBI Pathogen Isolates Browser at https://www.ncbi.nlm.nih.gov/pathogens/isolates/ (accessed on 1 June 2026).

## References

[1] Bhullar K., Waglechner N., Pawlowski A., Koteva K., Banks E.D., Johnston M.D., A Barton H., Wright G.D. (2012). Antibiotic Resistance is Prevalent in an Isolated Cave Microbiome. PLoS ONE.

[2] Ardakani Z., Canali M., Aragrande M., Tomassone L., Simoes M., Balzani A., Beber C.L. (2023). Evaluating the contribution of antimicrobial use in farmed animals to global antimicrobial resistance in humans. One Health.

[3] World Health Organization (WHO) (2019). No Time to Wait: Securing the Future from Drug-Resistant Infections.

[4] Martak D., Henriot C.P., Hocquet D. (2024). Environment, animals, and food as reservoirs of antibiotic-resistant bacteria for humans: One health or more?. Infect. Dis. Now.

[5] Castro-Vargas R.E., Herrera-Sánchez M.P., Rodríguez-Hernández R., Rondón-Barragán I.S. (2020). Antibiotic resistance in Salmonella spp. isolated from poultry: A global overview. Vet. World.

[6] Centers for Disease Control and Prevention (CDC) About *Salmonella* Infection. https://www.cdc.gov/salmonella/about/index.html.

[7] Punchihewage-Don A., Schwarz J., Diria A., Bowers J., Parveen S. (2024). Prevalence and antibiotic resistance of *Salmonella* in organic and non-organic chickens on the Eastern Shore of Maryland, USA. Front. Microbiol..

[8] Centers for Disease Control and Prevention (CDC) Chicken and Food Poisoning. https://www.cdc.gov/food-safety/foods/chicken.html.

[9] Wang J., Vaddu S., Bhumanapalli S., Mishra A., Applegate T., Singh M., Thippareddi H. (2023). A systematic review and meta-analysis of the sources of *Salmonella* in poultry production (pre-harvest) and their relative contributions to the microbial risk of poultry meat. Poult. Sci..

[10] Shaji S., Selvaraj R.K., Shanmugasundaram R. (2023). *Salmonella* Infection in Poultry: A Review on the Pathogen and Control Strategies. Microorganisms.

[11] Tilahun H.E., Efa D.A. (2026). Antimicrobial-resistant *Escherichia Coli* and *Salmonella* in poultry production and spread and effect in the one health framework. Poult. Sci..

[12] Zhou K., Sun L., Zhang X., Xu X., Mi K., Ma W., Zhang L., Huang L. (2023). *Salmonella* antimicrobials inherited and the non-inherited resistance: Mechanisms and alternative therapeutic strategies. Front. Microbiol..

[13] Brown E.W., Bell R., Zhang G., Timme R., Zheng J., Hammack T.S., Allard M.W. (2021). *Salmonella* Genomics in Public Health and Food Safety. EcoSal Plus.

[14] Hassanin A., Lories B., Steenackers H.P. (2025). *Salmonella* stress response systems as targets for anti-virulence strategies. BMC Microbiol..

[15] McDermott P.F., Tyson G.H., Kabera C., Chen Y., Li C., Folster J.P., Ayers S.L., Lam C., Tate H.P., Zhao S. (2016). Whole-Genome Sequencing for Detecting Antimicrobial Resistance in Nontyphoidal *Salmonella*. Antimicrob. Agents Chemother..

[16] Adesiji Y.O., Deekshit V.K., Karunasagar I. (2014). Antimicrobial-resistant genes associated with *Salmonella* spp. isolated from human, poultry, and seafood sources. Food Sci. Nutr..

[17] Sivanandy P., Yuk L.S., Yi C.S., Kaur I., Ern F.H.S., Manirajan P. (2024). A systematic review of recent outbreaks and the efficacy and safety of drugs approved for the treatment of *Salmonella* infections. IJID Reg..

[18] Lee K.Y., Atwill E.R., Pitesky M., Huang A., Lavelle K., Rickard M., Shafii M., Hung-Fan M., Li X. (2022). Antimicrobial Resistance Profiles of Non-typhoidal *Salmonella* from Retail Meat Products in California, 2018. Front. Microbiol..

[19] Brown A.C., Chen J.C., Watkins L.K.F., Campbell D., Folster J.P., Tate H., Wasilenko J., Van Tubbergen C., Friedman C.R. (2018). CTX-M-65 Extended-Spectrum β-Lactamase–Producing *Salmonella enterica* Serotype Infantis, United States1. Emerg. Infect. Dis..

[20] Karp B.E., Tate H., Plumblee J.R., Dessai U., Whichard J.M., Thacker E.L., Hale K.R., Wilson W., Friedman C.R., Griffin P.M. (2017). National Antimicrobial Resistance Monitoring System: Two Decades of Advancing Public Health Through Integrated Surveillance of Antimicrobial Resistance. Foodborne Pathog. Dis..

[21] Feldgarden M., Brover V., Gonzalez-Escalona N., Frye J.G., Haendiges J., Haft D.H., Hoffmann M., Pettengill J.B., Prasad A.B., Tillman G.E. (2021). AMRFinderPlus and the Reference Gene Catalog facilitate examination of the genomic links among antimicrobial resistance, stress response, and virulence. Sci. Rep..

[22] Neuert S., Nair S., Day M.R., Doumith M., Ashton P.M., Mellor K.C., Jenkins C., Hopkins K.L., Woodford N., de Pinna E. (2018). Prediction of Phenotypic Antimicrobial Resistance Profiles from Whole Genome Sequences of Non-typhoidal *Salmonella* enterica. Front. Microbiol..

[23] Cooper A.L., Low A.J., Koziol A.G., Thomas M.C., Leclair D., Tamber S., Wong A., Blais B.W., Carrillo C.D. (2020). Systematic Evaluation of Whole Genome Sequence-Based Predictions of *Salmonella* Serotype and Antimicrobial Resistance. Front. Microbiol..

[24] Zankari E., Hasman H., Cosentino S., Vestergaard M., Rasmussen S., Lund O., Aarestrup F.M., Larsen M.V. (2012). Identification of acquired antimicrobial resistance genes. J. Antimicrob. Chemother..

[25] Pettengill J.B., Tate H., Gensheimer K., Hsu C.H., Ihrie J., Markon A.O., Mcdermott P.F., Zhao S., Strain E., Bazaco M.C. (2020). Distribution of Antimicrobial Resistance Genes Across *Salmonella enterica* Isolates from Animal and Nonanimal Foods. J. Food Prot..

[26] Shen S., Fang F.C. (2012). Integrated stress responses in Salmonella. Int. J. Food Microbiol..

[27] Wang S., Phillippy A.M., Deng K., Rui X., Li Z., Tortorello M.L., Zhang W. (2010). Transcriptomic Responses of *Salmonella enterica* Serovars Enteritidis and Typhimurium to Chlorine-Based Oxidative Stress. Appl. Environ. Microbiol..

[28] Liu X., Omar M., Abrahante J.E., Nagaraja K.V., Vidovic S. (2020). Insights into the Oxidative Stress Response of *Salmonella enterica* serovar Enteritidis Revealed by the Next Generation Sequencing Approach. Antioxidants.

[29] Pei R., Zhang L., Duan C., Gao M., Feng R., Jia Q., Huang Z. (2021). Investigation of Stress Response Genes in Antimicrobial Resistant Pathogens Sampled from Five Countries. Processes.

[30] Li M., Wang K., Tang A., Tang A., Chen A., Huang Z. (2021). Investigation of the Genes Involved in the Outbreaks of *Escherichia coli* and *Salmonella* spp. in the United States. Antibiotics.

[31] Fàbrega A., Vila J. (2013). *Salmonella enterica* Serovar Typhimurium Skills To Succeed in the Host: Virulence and Regulation. Clin. Microbiol. Rev..

[32] de Oliveira S.D., Rodenbusch C.R., Michael G.B., Cardoso M.I., Canal C.W., Brandelli A. (2003). Detection of virulence genes in Salmonella Enteritidis isolated from different sources. Braz. J. Microbiol..

[33] Conner C.P., Heithoff D.M., Julio S.M., Sinsheimer R.L., Mahan M.J. (1998). Differential patterns of acquired virulence genes distinguish *Salmonella* strains. Proc. Natl. Acad. Sci. USA.

[34] Webber B., Borges K.A., Furian T.Q., Rizzo N.N., Tondo E.C., dos Santos L.R., Rodrigues L.B., Nascimento V.P.D. (2019). Detection of virulence genes in Salmonella Heidelberg isolated from chicken carcasses. Rev. Inst. Med. Trop. São Paulo.

[35] Dione M.M., Ikumapayi U., Saha D., Mohammed N.I., A Adegbola R., Geerts S., Ieven M., Antonio M. (2011). Antimicrobial resistance and virulence genes of non-typhoidal Salmonella isolates in The Gambia and Senegal. J. Infect. Dev. Ctries..

[36] Cui K., Gong I., Dong A., Yan J., Wang M., Huang Z. (2020). Investigation of Virulence Genes Detected in Antimicrobial-Resistance Pathogens Isolates for Five Countries across the World. Processes.

[37] Han J., Aljahdali N., Zhao S., Tang H., Harbottle H., Hoffmann M., Frye J.G., Foley S.L. (2024). Infection biology of *Salmonella enterica*. EcoSal Plus.

[38] Han J., Tang E., Zhao S., Foley S.L. (2026). Genomic characterization of antimicrobial resistance and virulence determinants in *Salmonella* Infantis isolated from human, food, and animal sources. Appl. Environ. Microbiol..

[39] Nuanmuang N., Leekitcharoenphon P., Njage P.M.K., Gmeiner A., Aarestrup F.M. (2023). An Overview of Antimicrobial Resistance Profiles of Publicly Available *Salmonella* Genomes with Sufficient Quality and Metadata. Foodborne Pathog. Dis..

[40] Guard J., Cao G., Luo Y., Baugher J.D., Davison S., Yao K., Hoffmann M., Zhang G., Likens N., Bell R.L. (2020). Genome sequence analysis of 91 *Salmonella* Enteritidis isolates from mice caught on poultry farms in the mid 1990s. Genomics.

[41] Wijesena H.R., Harhay D.M., Katz T.S., Wheeler T.L., Schmidt J.W., Arthur T.M., Bono J.L. (2026). A Computational Protocol for Enhanced Characterization of Sequence Differences Among *Salmonella enterica* Isolates within SNP Clusters Identified in the NCBI Pathogen Detection System. J. Food Prot..

[42] Jolliffe I.T., Cadima J. (2016). Principal component analysis: A review and recent developments. Philos. Trans. R. Soc. A Math. Phys. Eng. Sci..

[43] Joliffe I., Morgan B. (1992). Principal component analysis and exploratory factor analysis. Stat. Methods Med. Res..

[44] Hotelling H. (1933). Analysis of a complex of statistical variables into principal components. J. Educ. Psychol..

[45] Rafsanjani M.K., Varzaneh Z.A., Chukanlo N.E. (2012). A Survey of Hierarchical Clustering Algorithms. J. Math. Comput. Sci..

[46] Jafarzadegan M., Safi-Esfahani F., Beheshti Z. (2019). Combining hierarchical clustering approaches using the PCA method. Expert Syst. Appl..

[47] Murtagh F., Legendre P. (2014). Ward’s Hierarchical Agglomerative Clustering Method: Which Algorithms Implement Ward’s Criterion?. J. Classif..

[48] Panda N.R. (2022). A Review on Logistic Regression in Medical Research. Natl. J. Community Med..

[49] Boateng Y., Abaye D.A. (2019). A Review of the Logistic Regression Model with Emphasis on Medical Research. J. Data Anal. Inf. Process..

[50] Kim H.-Y. (2017). Statistical notes for clinical researchers: Chi-squared test and Fisher’s exact test. Restor. Dent. Endod..

[51] Green G.H., Diggle P.J. (2007). On the Operational Characteristics of the Benjamini and Hochberg False Discovery Rate Procedure. Stat. Appl. Genet. Mol. Biol..

[52] Genovese C., Wasserman L. (2002). Operating Characteristics and Extensions of the False Discovery Rate Procedure. J. R. Stat. Soc. Ser. B Stat. Methodol..

[53] Hastie T., Tibshirani R., Friedman J. (2009). The Elements of Statistical Learning: Data Mining, Inference, and Prediction.

[54] Eisen M.B., Spellman P.T., Brown P.O., Botstein D. (1998). Cluster analysis and display of genome-wide expression patterns. Proc. Natl. Acad. Sci. USA.

[55] Fawcett T. (2006). An Introduction to ROC analysis. Pattern Recogn. Lett..

[56] Benjamini Y., Hochberg Y. (1995). Controlling the False Discovery Rate: A Practical and Powerful Approach to Multiple Testing. J. R. Stat. Soc. Ser. B Methodol..

[57] Tukey J.W. (1977). Exploratory Data Analysis.

[58] Scott A.J., Hosmer D.W., Lemeshow S. (1991). Applied Logistic Regression. Biometrics.

[59] Mensah N., Tang Y., Cawthraw S., AbuOun M., Fenner J., Thomson N.R., Mather A.E., Petrovska-Holmes L. (2019). Determining antimicrobial susceptibility in *Salmonella enterica* serovar Typhimurium through whole genome sequencing: A comparison against multiple phenotypic susceptibility testing methods. BMC Microbiol..

[60] Chiou C.-S., Jones A. (1995). Expression and identification of the strA-strB gene pair from streptomycin-resistant Erwinia amylovora. Gene.

[61] Monte D.F.M., Harrell E., Harden L., Thakur S. (2025). Clonal spread of bla_CTX-M-65_ producing *Salmonella enterica* serovars detected in poultry retail meat in North Carolina, USA. Sci. Rep..

[62] Cooper A.L., Wong A., Tamber S., Blais B.W., Carrillo C.D. (2024). Analysis of Antimicrobial Resistance in Bacterial Pathogens Recovered from Food and Human Sources: Insights from 639,087 Bacterial Whole-Genome Sequences in the NCBI Pathogen Detection Database. Microorganisms.

[63] Song S., Lee B., Yeom J.-H., Hwang S., Kang I., Cho J.-C., Ha N.-C., Bae J., Lee K., Kim Y.-H. (2015). MdsABC-Mediated Pathway for Pathogenicity in *Salmonella enterica* Serovar Typhimurium. Infect. Immun..

[64] Feldgarden M., Brover V., Fedorov B., Haft D.H., Prasad A.B., Klimke W. (2022). Curation of the AMRFinderPlus databases: Applications, functionality and impact. Microb. Genom..

[65] Lee W.W.Y., Mattock J., Greig D.R., Langridge G.C., Baker D., Bloomfield S., Mather A.E., Wain J.R., Edwards A.M., Hartman H. (2021). Characterization of a pESI-like plasmid and analysis of multidrug-resistant *Salmonella enterica* Infantis isolates in England and Wales. Microb. Genom..

[66] Benefo E.O., Ramachandran P., Pradhan A.K. (2024). Genome-based machine learning for predicting antimicrobial resistance in *Salmonella* isolated from chicken. LWT.

[67] Fenske G.J., Scaria J. (2021). Analysis of 56,348 Genomes Identifies the Relationship between Antibiotic and Metal Resistance and the Spread of Multidrug-Resistant Non-Typhoidal Salmonella. Microorganisms.

[68] Hikal A.F., Hasan S., Gudeta D., Zhao S., Foley S., Khan A.A. (2024). The acquired pco gene cluster in *Salmonella enterica* mediates resistance to copper. Front. Microbiol..

[69] Chen S., Fu J., Zhao K., Yang S., Li C., Penttinen P., Ao X., Liu A., Hu K., Li J. (2023). Class 1 integron carrying qacEΔ1 gene confers resistance to disinfectant and antibiotics in Salmonella. Int. J. Food Microbiol..

[70] Aviv G., Tsyba K., Steck N., Salmon-Divon M., Cornelius A., Rahav G., Grassl G.A., Gal-Mor O. (2014). A unique megaplasmid contributes to stress tolerance and pathogenicity of an emergent *Salmonella enterica* serovar Infantis strain. Environ. Microbiol..

[71] Mustafa G.R., Zhao K., He X., Chen S., Liu S., Mustafa A., He L., Yang Y., Yu X., Penttinen P. (2021). Heavy Metal Resistance in *Salmonella* Typhimurium and Its Association with Disinfectant and Antibiotic Resistance. Front. Microbiol..

[72] Mattock J., Chattaway M.A., Hartman H., Dallman T.J., Smith A.M., Keddy K., Petrovska L., Manners E.J., Duze S.T., Smouse S. (2024). A One Health Perspective on *Salmonellaenterica* Serovar Infantis, an Emerging Human Multidrug-Resistant Pathogen. Emerg. Infect. Dis..

[73] Zwe Y.H., Chin S.F., Kohli G.S., Aung K.T., Yang L., Yuk H.-G. (2020). Whole genome sequencing (WGS) fails to detect antimicrobial resistance (AMR) from heteroresistant subpopulation of *Salmonella enterica*. Food Microbiol..

